# Age-dependent predictors of effective reinforcement motor learning across childhood

**DOI:** 10.1101/2024.07.09.602665

**Published:** 2024-07-09

**Authors:** Nayo M. Hill, Haley M. Tripp, Daniel M. Wolpert, Laura A. Malone, Amy J. Bastian

**Affiliations:** 1 Kennedy Krieger Institute, Baltimore, MD; 2 Department of Neuroscience, Johns Hopkins School of Medicine, Baltimore, MD; 3 Mortimer B. Zuckerman Mind Brain Behavior Institute, Columbia University, New York, NY; 4 Department of Neuroscience, Columbia University, New York, NY; 5 Department of Neurology, Johns Hopkins School of Medicine, Baltimore, MD; 6 Department of Physical Medicine and Rehabilitation, Johns Hopkins School of Medicine, Baltimore, MD

## Abstract

Across development, children must learn motor skills such as eating with a spoon and drawing with a crayon. Reinforcement learning, driven by success and failure, is fundamental to such sensorimotor learning. It typically requires a child to explore movement options along a continuum (grip location on a crayon) and learn from probabilistic rewards (whether the crayon draws or breaks). Here, we studied the development of reinforcement motor learning using online motor tasks to engage children aged 3 to 17 and adults (cross-sectional sample, N=385). Participants moved a cartoon penguin across a scene and were rewarded (animated cartoon clip) based on their final movement position. Learning followed a clear developmental trajectory when participants could choose to move anywhere along a continuum and the reward probability depended on final movement position. Learning was incomplete or absent in 3 to 8-year-olds and gradually improved to adult-like levels by adolescence. A reinforcement learning model fit to each participant identified three age-dependent factors underlying improvement: amount of exploration after a failed movement, learning rate, and level of motor noise. We predicted, and confirmed, that switching to discrete targets and deterministic reward would improve 3 to 8-year-olds’ learning to adult-like levels by increasing exploration after failed movements. Overall, we show a robust developmental trajectory of reinforcement motor learning abilities under ecologically relevant conditions i.e., continuous movement options mapped to probabilistic reward. This learning appears to be limited by immature spatial processing and probabilistic reasoning abilities in young children and can be rescued by reducing the demands in these domains.

## Introduction

In the game of Poohsticks, invented by A. A. Milne and described in *The House at Pooh Corner*, two children each drop a stick into a stream from the upstream side of a bridge [1]. They then race to the downstream side to see whose stick appears first, with the winner scoring a point. The game is repeated with each child trying to find the sweet spot to drop their stick in to win. Given the capricious nature of streams with their turbulent flow, dropping both sticks in exactly the same spot on two successive games can lead to different outcomes. To be an expert, a child must use probabilistic success and failure feedback to select a location from the infinite options available (the continuous span of the bridge) to drop their stick to maximize reward. Despite the complexity of this probabilistic reinforcement task, the current world champion is 9 years old. Here we examine how children develop the ability to learn such tasks from reinforcement feedback alone.

Reinforcement learning is essential for successful movement. Unlike error-based learning that uses a vector error, reinforcement learning relies on reward feedback that is indicated by a scalar value. Reward feedback can be simple binary feedback, such as success or failure in hitting the space bar on a keyboard, or continuous, such as the height achieved on a swing. Critically, the learner is not told what to do but must discover which movement or behavior produces reward by trying different options [2]. Therefore, a key component of reinforcement learning is exploring and evaluating feedback to maximize reward.

The basic capacity for reinforcement learning emerges early in life. For example, a nine-week-old infant will increase kicking frequency when a ribbon connects their foot to an overhead mobile that moves with their kicks [3]. Three-month-olds can learn to produce a specific kick height to move a mobile [4]. Both tasks have a deterministic relationship between the action and the outcome; a correct kick is always rewarded. In a more complex probabilistic reaching task, children aged 3- to 9-years old showed different sensitives to reward probability [5]. The youngest aged children were more likely to stick on a rewarded target even if the reward rate was low (e.g., 33%), whereas older children explored other targets. This suggests that younger children are less likely to explore new options and are more willing to accept lower reward rates.

Reinforcement learning has been studied in cognitive tasks that require children to select from discrete options to receive reward. In a relatively simple task, two-year old children could accurately select a reinforced visual image from two choices [6]. More complex tasks have been studied in older children and adolescents identifying age-related changes in learning ability [7–11]. In one task, participants selected from two options in a probabilistic reward environment with hidden factors that could change the outcome. Unbeknownst to the participants, agents (e.g., millionaires, robbers, or sheriffs) occasionally intervened to generate positive, negative, or random outcomes [8]. Both children and adolescents could identify the factors in the environment that changed the outcome. However, children under 12 years old did not use this information; younger children’s reinforcement learning mechanism was unable to use declarative information to optimize their choices. Similarly, Decker et al. used a sequential learning task with probabilistic rewards to show that probabilistic reasoning improves with age in a cohort of participants aged 8 to 25 years [9]. As a whole, this previous work identifies differences between younger and older children on choice-based selection tasks that require integration of reward feedback to learn successfully.

Although motor learning tasks have cognitive aspects [12], they also have features which are not present in such cognitive tasks. In motor learning, movement choice options are typically continuous (the choice of direction to kick a soccer ball), corrupted by motor noise, and there can be spatial gradient of reward (the probability of scoring as a function of kick direction). In contrast, cognitive tasks tend to be discrete with arbitrary assignment of reward probability to options. Here, we examine motor learning under reinforcement in which we control two key experimental factors. First, rewards can either be deterministic, the same action leads to the same outcome (e.g., pressing the space bar on a keyboard), or probabilistic, the outcome is stochastic (e.g., the path of a soccer ball depends on the wind and surface on which it is kicked). Second, action options can be discrete (e.g., the space or shift key on a keyboard) or continuous (e.g., the direction of a soccer kick). We report on a series of experiments in which we control both factors, reinforcement feedback (deterministic vs. probabilistic) and the action options (discrete vs. continuous targets) to examine the development of reinforcement learning across childhood. Our study builds on previous work in healthy adults examining reaching movements under binary reward feedback [13, 14].

We developed a remote video game paradigm for a cross-sectional study of 298 children aged 3 to 17 years and 87 adults from across the USA (locations and demographics shown in Supp. Fig. 1 and Table 1). Focusing on children’s reinforcement learning abilities in different tasks, we examined the developmental trajectory of exploration variability, learning rate, and motor noise. We found that younger children (3 to 8 years) fail to learn with a continuous target and probabilistic reward feedback. Reinforcement learning improved with age enabling older children to find the optimal reward region. Using a mechanistic model, we show that this improvement is due to a developmental gradient of increasing exploration, increasing learning rate, and reducing motor noise with age. Importantly, we then show that use of deterministic feedback and discrete targets can dramatically improve learning abilities of younger children.

## Results

**Fig 1.**
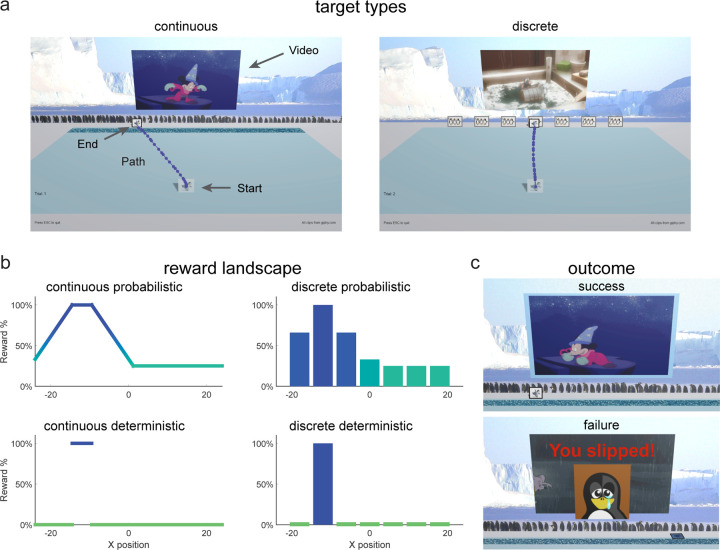
Game Environment. **a.** Screenshot of game environment and sample movement path (large text, arrows, and movement path were not displayed to participants). During the learning block, participants either experienced a continuous target (continuous groups) or seven discrete targets (discrete groups). **b.** Reward landscape for the learning blocks for the different task paradigms. Continuous probabilistic: continuous target with position-based reward probability gradient; discrete probabilistic: discrete targets with target specific reward probabilities; continuous deterministic: continuous target with a single 100% rewarded zone; discrete deterministic: discrete targets with a single target giving 100% reward. **c.** Outcome feedback for continuous probabilistic task. Success (top), movie clip and pleasant sound plays, and video screen is outlined in blue. Failure (bottom), movie clip does not play, the penguin falls over and red text “You slipped!” appears with a sad face image.

In the first experiment (continuous probabilistic task), we studied 111 children and adolescents, aged 3 to 17 years, and 33 adults as they attempted to learn to produce movements that maximized reward. For the key learning phase of the experiment, we created a probabilistic reward landscape in which the probability of reward after each movement depended on its endpoint. To implement this in a task that was engaging to children, we created a computer game that was played at home. Participants were required to move a cartoon penguin from a starting position to join a group of penguins arranged horizontally across the screen (Fig. 1a - continuous). Participants were told that there was a slippery ice patch just before the group (dark blue area, Fig. 1a - continuous) and that they should try to make the penguin cross at the location where they would not slip. In the instructions, participants were told that there was a location in the ice where they would never slip. The reward landscape (Fig. 1b top left) determined the probability of success on each trial and was not known to the participant. There was a small 100% reward zone where the penguin would never slip with the reward probability decreasing away from this zone. Successful trials led to a short Disney cartoon clip playing whereas a failure trial led to the penguin falling over and the appearance of a static image of a sad penguin (Fig. 1c).

**Fig 2.**
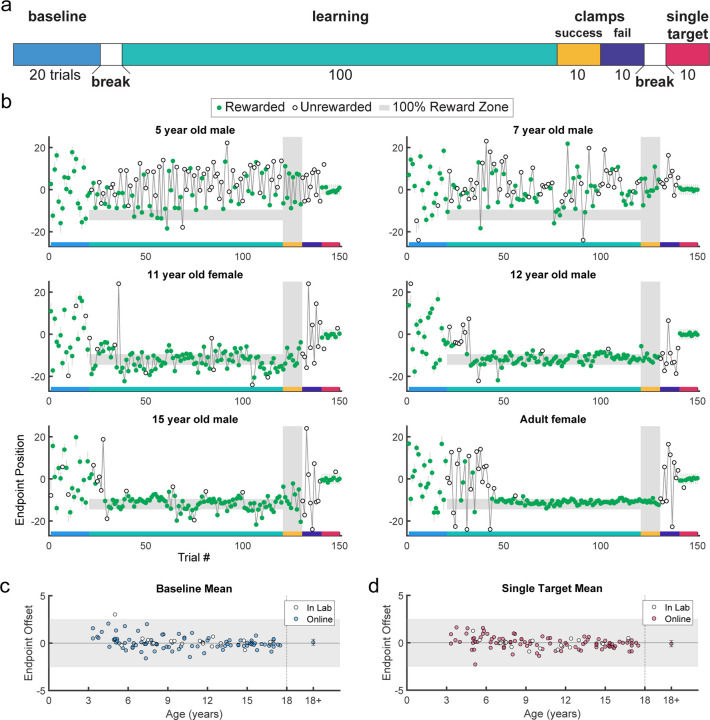
Paradigm, example behavior, and target accuracy in the continuous probabilistic task. **a.** Experimental design with baseline: single discrete target presented in randomized locations across the screen; learning: learning block with reward determined by endpoint position; success clamp: feedback clamped to 100% success independent of endpoint position; fail clamp: feedback clamped to 100% failure independent of endpoint position; single target: single discrete target presented in the middle of the screen. **b.** Representative endpoint time series from various aged participants. Gray shaded zones indicate positions in the workspace where a reward is given 100% of the time (thin gray lines are for discrete targets). Green filled circles indicate rewarded trials while open circles indicate unrewarded trials. The horizontal colored bar on the x-axis indicates the trials corresponding with the experimental blocks outlined in **a**. In the learning block (trials 21–120), rewards were given based on the continuous probabilistic landscape. **c.** Mean baseline accuracy (average reach deviation from the discrete targets) by age. Adult data are averaged and plotted to the right with standard error of the mean. The gray region shows the width of a discrete target. **d.** Same as **c** for the single target in block 5. In **c** and **d**, participants who completed the task in person (in lab) are indicated in white circles.

Participants performed 5 blocks of trials (Fig. 2a). In block 1, participants made 20 baseline movements to a series of small, discrete targets that appeared at different locations (sample paths are shown in Supp. Figs. 2 and 3). The targets were presented in a randomized order but each participant received the same 20 target locations. This allowed the participants to experience reaching to many locations in the workspace as well as assessed their accuracy to hit discrete targets. Fig. 2b shows examples from different aged participants. All performed well in the baseline block of the task (trials 1–20). On average, all but one participant could accurately hit targets (Fig. 2c) although younger children tended to make less straight movements (Supp. Fig. 4) and took longer to initiate and start trials (Supp. Fig. 5). Participants then began the learning block (described above), where they could move to any endpoint location on the screen. Fig. 2b shows representative examples of endpoint locations across the experiment for participants of different ages. The 5- and 7-year-old children moved to many endpoint locations (i.e., showed exploration) receiving reward based on the probability landscape. Interestingly, their exploration appeared to show little sensitivity to previous failure (open circles) versus success (filled circles) and endpoints did not converge on the 100% reward zone (gray area) by the end of the block. The older children, aged 11, 12, and 15, performed similarly to the adult, exploring early in the block, and then focusing movements on the unseen 100% reward zone. This indicates that they were able to explore successfully after failures and exploit the 100% reward zone.

**Fig 3.**
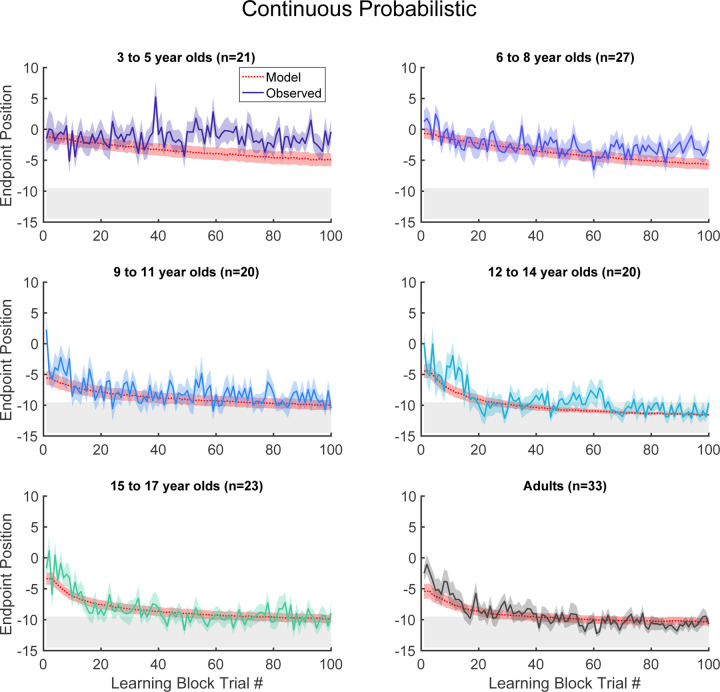
Continuous probabilistic task learning block time series. Data and model (red, smooth curves) for each trial of the learning block grouped into age ranges. Data shows mean (solid line) and standard error of mean (shading) of participants’ endpoint. Model in red shows mean (dashed lines) and standard deviation (shading) from the model simulations. The gray region shows 100% reward zone.

These patterns were also observed in group data (Fig. 3, binned across 6 age groups) where, on average, the 3 to 5 and 6 to 8-year-olds did not find the 100% reward zone, but the older age groups did. After learning, participants transitioned seamlessly (no break or change in instructions) into two short blocks with clamped feedback to examine behavior in response to repeated success or repeated failure. The success clamp always rewarded movements and the fail clamp never rewarded movements. The 5- and 7-year-olds explored similarly in both clamped blocks whereas the older children showed greater exploration in the fail clamp compared to the success clamp (Fig. 2b). In the final block, participants moved to a single discrete target in the center of the workspace (examples Fig. 2b; group data Fig. 2d). For participants age 3 to 17 years, an ANOVA of accuracy (distance from discrete target center) by block (first vs. last) and age (5 bins) shows no effect of block (*F*_1,106_ = 0.282, p = 0.597) and no interaction between age and block (*F*_4,106_ = 1.219, p = 0.307), indicating that accuracy of movement was maintained throughout the experiment even for the younger children.

**Fig 4.**
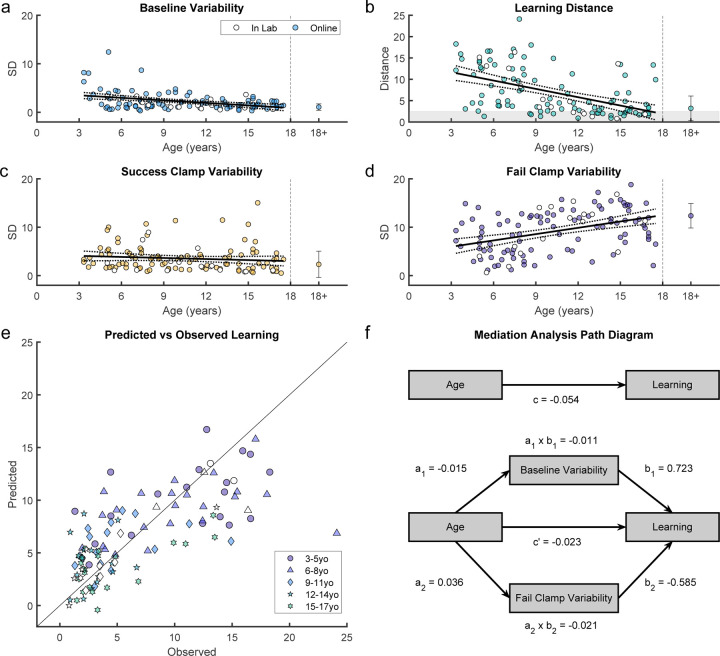
Variability and learning in the continuous probabilistic task. **a.** Baseline variability by age. Average adult variability shown for comparison. **b.** Learning block performance (absolute distance from 100% reward zone) by age. **c.** Endpoint variability in the success clamp by age. **d.** Endpoint variability in the failure clamp by age. For **a** - **d**, regression line with 95% confidence limits shown for children and error bars show standard error of the mean for adults. **e.** Predicted vs. observed performance from the multiple regression of learning as a function of age, baseline variability and fail clamp variability. **f.** Mediation analysis (see Methods for details). Top pathway shows the direct relationship between age and learning. Bottom pathway shows the indirect relationship between age and learning when mediated by baseline variability and fail clamp variability. Note that in our measure of learning, smaller distances from the 100% reward zone reflect better learning, which explains the negative relationships in this analysis (e.g., increasing age is associated with decreased distances from the reward zone). Age is coded in months. Participants who completed the task in person (in lab) are indicated in white symbols.

We assessed key features of behavior in the first four blocks of the experiment that could contribute to the learning patterns observed across age (Fig. 4a–d, with the average of adult performance for comparison). In the Baseline block, although all participants were able to hit the targets, the endpoint variability relative to target center decreased significantly with age (Fig. 4a; regression *R*^2^ = 0.153, *F*_1,109_ = 19.7, p < 0.001). This is consistent with known developmental changes in motor variability [15, 16] and may represent motor noise which is distinct from exploration.

We quantified final performance in the learning block as the distance of the reaches from the center of the 100% reward zone averaged over the last 10 trials of the block. In children, the distance significantly reduced with age indicating better learning (Fig. 4b; regression *R*^2^ = 0.256, *F*_1,109_ = 37.4, p < 0.001). Younger children rarely reached the 100% reward zone whereas children over 9 years old often did.

To be successful in the task, we would expect participants to behave differently after a failure versus after a success. That is, participants need to explore to find the 100% reward zone when their reward rate is low (i.e., after failure) and reduce exploration when their reward rate is high (i.e., after success). We used the clamp blocks to assess how participants responded to repeated success versus repeated failure, and if this contributed to developing adult-like learning patterns. We calculated the standard deviation of movement endpoints in each clamp condition as a measure of movement variability (Fig. 4c & d). Adults showed high variability after failure and low variability after success, as expected. In children, variability after failure was low in younger children and increased significantly with age (regression *R*^2^ = 0.17, *F*_1,109_ = 22.3, p < 0.001) to reach adult levels. Regression shows that variability after failure doubles across the age range. This likely reflects an increase in active exploration in an attempt to find the reward zone. In contrast, variability after success was relatively stable across ages (regression *R*^2^ = 0.012, *F*_1,109_ = 1.34, p = 0.249). Overall, these results suggest that younger children do not explore as much as older children after failures, which is essential to finding the 100% reward zone.

We hypothesized that a combination of age and the variability in the baseline, success clamp, and fail clamp blocks could be predictors of final performance. Using multiple linear regression, we found that three of the four factors were significant predictors of learning (*R*^2^ = 0.537, *F*_4,106_ = 30.7, p < 0.001). Variability after failure was the strongest predictor of learning (*β* = −0.613, p < 0.001) followed by baseline variability (*β* = 0.706, p = 0.001) and age (*β* = −0.019, p = 0.022). Variability after success was not significant (*β* = 0.244, p = 0.067). Fig. 4e shows that a model with the three significant regressors predicts the observed behavior well (*R*^2^ = 0.703, p < 0.001). We then performed a mediation analysis to determine if variability in the baseline and fail clamp blocks mediated the effect of age on learning (Supplementary Table 1). Both mediators significantly reduced, but did not eliminate, the direct effect of age on learning (Fig. 4f, *c*^′^ was reduced compared to *c* in the mediation, but still remained significant. See Methods for details). Thus, baseline variability and variability after failure can explain part of the effect of age on learning, but age still has a significant direct effect.

Up to this point, we have considered variability in the baseline and clamp blocks as a combination of sensorimotor noise and exploration. To separate motor noise from exploration and to understand how experience changes behavior, we developed a reinforcement learning model of the task. To determine the structure of the model, we first examined how many recent trials affected the current trial. Specifically, we considered how failures up to three trials back affected how participants change their current endpoint (See Methods for additional details). This analysis (Supp. Fig. 6) showed that for the majority of participants only the just previous failure trial impacted the change in endpoint on the current trial and that failures further back did not affect current behavior. Given this, the reinforcement learning model assumes that exploration only depends on the outcome of the previous trial.

**Fig 5.**
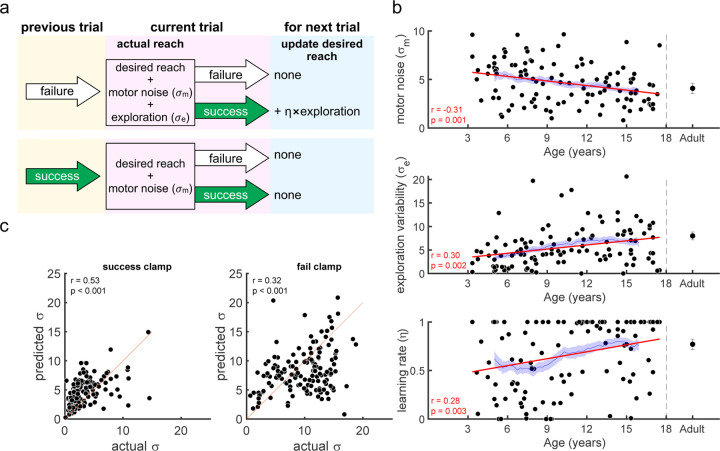
Reinforcement learning model for the continuous probabilistic task. **a.** Model schematic. The participant maintains an estimate of desired reach which they can update across the experiment. The actual reach on the current trial (pink box) depends on whether the previous trial (yellow box) was a failure (top) or success (bottom). After failure (top) the actual reach is the desired reach with the addition of exploration and motor noise (draws from zero mean Gaussian distributions with standard deviations $\sigma_{e}$ and $\sigma_{m}$, respectively). In contrast, if the previous trial was a success (bottom), the participant does not explore so that the actual reach is the desired reach with only motor noise. The actual reach determines the probability of whether the current trial is a failure or a success. If the current trial is a success the desired reach is updated for the next trial (blue box) by the exploration (if any), modulated by a learning rate η. **b.** Model fit parameters $\left\{\sigma_{m},\sigma_{e},\eta \right\}$, by age for the continuous probabilistic group. The solid thick line is a regression fit to the data for participants less than 18 years old and the thin line is a running mean ±3 years with the standard error of the mean. The correlation and p-value for each regression are shown in the bottom left corner of each plot (and exclude the adult data). Average adult parameters are shown on the right with standard error of the mean. **c.** Predicted vs. actual variability in the success (left column) and fail (right column) clamp blocks. Correlations and p-vales are shown above each plot (the plots and statistics exclude the adult data).

We modeled each participant as a learner who maintains an estimate of their desired reach location which can be updated. If the previous trial was a failure (Fig. 5a, top), the current reach is the desired reach with the addition of motor noise and exploration (draws from zero mean Gaussian distributions with standard deviations $\sigma_{e}$ and $\sigma_{m}$, respectively). The desired reach for the next trial is updated only if the current reach is successful. The update is a proportion (η, termed the learning rate) of the exploration that led to success. In contrast, if the previous reach was a success (Fig. 5a, bottom) the participant does not explore so that the current reach is the desired reach with motor noise. As there is no exploration, the desired reach will remain the same for the next trial whatever the outcome (see Methods for details).

We fit the model to the 100 learning trials for each child and adult participant (see Methods for details), so as not to contaminate the fits by behavior in the clamp blocks. We first examined how well our model fitting procedure could recover parameters from synthetic data. This showed that all three parameters ($\sigma_{m}$*, $\sigma_{e}$,η*) were well recovered with correlations with the true parameters of at least 0.99 (Supp. Fig. 7). We examined how the three parameters fit to the actual data varied with age (Fig. 5b). This showed that all three parameters varied significantly with age for the children (all p < 0.01). Motor noise decreased by about 40% within the age range tested consistent with our measure of baseline variability which also decreased significantly with age. Exploration variability increased with age, approximately doubling on average from the youngest to the oldest child. While this is similar to the variability findings in the fail clamp, the model allows us to examine exploration variability in the learning setting and separate it from motor noise. In addition to exploration increasing with age, the learning rate also increased across the age span from about 0.5 to around 0.8 by 17 years. Overall, this suggests that the increased motor noise, reduced exploration, and reduced learning rate limits the ability of the younger children to learn efficiently.

The simulations of the model with the fit parameters accounted for the differences in learning across the age group bins (Fig. 3 red dotted line and shading show model simulations). The model simulated data performed similarly to the actual data from children age 6 and older as well as the adults. Note that the model simulated data performed slightly better than the youngest children. The model also makes a prediction of the reach variability expected in the two clamp trial blocks. In the success clamp block the variability (s.d.) only arises from motor noise and should be $\sigma_{m}$. For the fail clamp block it arises from both motor noise and exploration and should be $\sqrt{\sigma_{e}^{2}+\sigma_{m}^{2}}$. Fig. 5c shows the variability expected from the model against the measured variability of the children in the clamp blocks (left and right columns for success and fail clamps). These out-of-sample predictions showed significant positive correlations for both the success and fail clamp blocks (p < 0.001). This was despite having only 9 trials to calculate each clamp block s.d., suggesting that the model fit to learning can predict differences in exploration.

Poor or absent learning in the youngest age group could arise from these participants not understanding the task. However, we can rule out task comprehension as an explanation for lack of learning, at least for the majority of younger participants. First, 16 participants completed the task in the lab, and this sample included 5 participants in the 3- to 8-year-old age range. The task was administered in the same way as the online version, without additional instructions or interaction with the experimenter during the task. However, after completion, the experimenter asked these children to explain what they did during the task as an informal comprehension check and it was clear that this group understood the task. Performance for all participants tested in the lab was not significantly different from online completion and these data points are indicated in Fig. 2 and Fig. 4 with white symbols. Wilcoxon signed-rank tests showed no significant difference between in lab and online participants in baseline mean (Z = 1.238, p = 0.216), baseline variability (Z = −0.626, p = 0.532), learning distance (Z = −0.348, p = 0.728), success variability (Z = −1.952, p = 0.051), fail clamp variability (Z = 0.281, p = 0.779), or single target mean (Z = −0.399, p = 0.690).

Second, we used the reinforcement learning model to assess whether individual’s performance is consistent with understanding the task. In the case of a child who does not understand the task, we expect that they simply have motor noise on their reach, but crucially that they would not explore more after failure, nor update their reach after success. Therefore, we fit both a reduced model (motor noise only) as well as the full model (including exploration and learning) to each participant. We used a likelihood ratio test to examine whether the full model was significantly better at explaining each participant’s data compared to the reduced, non-learning model. Importantly, even if there is no learning on average, the full model can capture aspects of the data. For example, increased exploration after failure with a learning rate of zero would give no learning but the full model could explain the data better as it would capture the changes in variance as a function of trial success or failure. Even with a non-zero learning rate the model can show little overall learning but capture aspects of the child’s behavior over the learning block. This analysis shows that only 19 of the 144 participants were better fit with the noise only model with the remaining participants best fit with the full model. Importantly, 39 out of 48 of the younger participants (3 to 8 years) were better fit by the full model, indicating non-zero exploration after failure and typically a non-zero learning rate. Supp. Fig. 8 shows averages for those fit best by the motor noise model (left) and those fit best by the full model (right). The younger children who are best fit by the full model show minimal learning, similar to what we report for the entire group (Fig. 3). Therefore, our results are robust to only including participants whose data is best accounted for by including exploration and learning.

Thus far, we identified features that contribute to the younger children’s inability to learn within a continuous probabilistic landscape. Specifically, less variability after failure, more baseline variability, and younger age. In a second experiment, we asked if segmenting the continuous target into seven discrete targets could improve learning (Fig. 1a - discrete). In this new task, participants were not able to move between targets but were required to definitively select a single target on each trial in order for the experiment to advance. We hypothesized that discrete targets could increase exploration by encouraging children to move to a different target after failure. We studied a new cohort of 106 children and 33 adults as they learned the discrete probabilistic task (Fig. 1b top right). Supp. Fig. 9 shows individual example data. Some of our participants tended to move to a new target after failure (e.g., the 6 and 16 year old). Group data (Supp. Fig. 10) showed modest benefit in the 3 to 5 and 6 to 8 year-old participants, with all other age groups performing similarly compared to the continuous task. Supp. Fig. 11 shows variability and learning measures which are similar to the continuous group. Learning distance decreased with age (regression *R*^2^ = 0.074, *F*_1,104_ = 8.31, p = 0.0048) indicating better learning performance. Baseline endpoint variability and variability after success decreased with age (baseline: regression *R*^2^ = 0.150, *F*_1,104_ = 18.4, p < 0.001; success clamp: regression *R*^2^ = 0.048, *F*_1,104_ = 5.25, p = 0.024) while variability after failure increased with age (regression *R*^2^ = 0.162, *F*_1,104_ = 20.1, p < 0.001).

We performed multiple regression, as for the continuous task, which showed that only variability after failure (*β* = −0.343, p < 0.001) and after success (*β* = 1.171, p < 0.001) but not age (*β* = −0.002, p = 0.722) or baseline variability (*β* = −0.210, p = 0.070) were significant predictors of learning (*R*^2^ = 0.764, *F*_4,101_ = 81.9, p < 0.001). Supp. Fig. 11e shows that the model with only the significant regressors predicts the learning well (*R*^2^ = 0.686, p < 0.001). In contrast to the continuous task, baseline variability did not affect learning. This is likely because in the discrete task, as long as you can reach to your target of choice, motor noise will not affect the outcome. In order to understand why variability after success and failure, but not age, were significant regressors, we performed a mediation analysis (Supp. Fig. 11f). This showed that although age alone was a predictor of learning, it was fully mediated by variability after success and failure (Supplementary Table 2).

We used the reinforcement model to fit data in the discrete probabilistic task. The model is fit to the reach endpoint locations and rewards the participants received (that is we do not explicitly represent discrete targets in the model). Compared to the continuous task, the parameters did not change significantly with age (Supp. Fig. 12). To compare the fit parameter by age across the two tasks, we compared the linear regression. This showed that the exploration and learning rate had higher intercepts for the discrete task (both p < 0.05) but there was no significant difference in motor noise. This suggests that the discrete nature of the task encouraged all participants to explore more and update their desired reach more after success.

**Fig 6.**
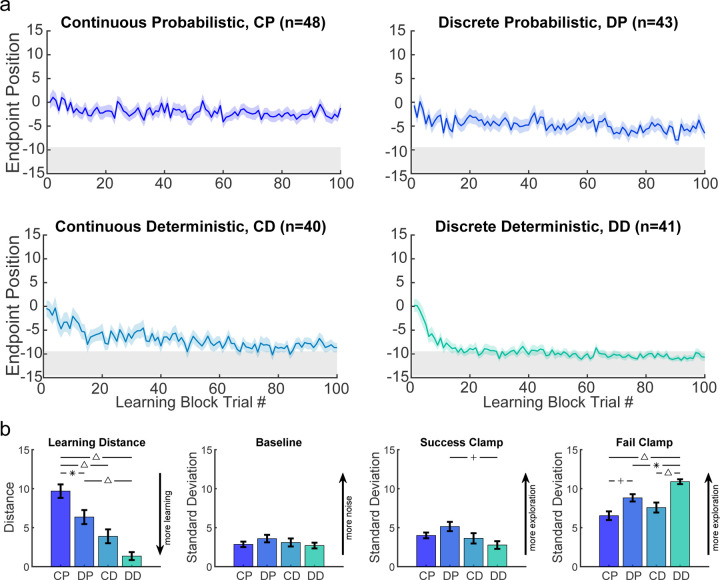
Comparison of the four tasks for the three- to eight-year-old children. **a.** Learning block performance for the continuous probabilistic, discrete probabilistic, continuous deterministic, and discrete deterministic tasks in the same format as Fig. 3. **b.** Comparative performance between tasks for learning distance and variability in baseline, success clamp, and fail clamp. Learning significantly improved with discrete targets and deterministic reward feedback. Baseline variability was not statistically different between tasks. Statistically significant pairwise comparisons indicated as follows: * = p < 0.05, + = p < 0.01, and ∆ = p < 0.001. Abbreviations: CD = Continuous Deterministic; CP = Continuous Probabilistic; DD = Discrete Deterministic; DP = Discrete Probabilistic

In both experiments considered so far, the reward was probabilistic. While this provides gradient information that should guide the participant to the target, younger children may not use the same strategy for this reward landscape. In the final two tasks, we studied new cohorts of 3–8-year-old children since they showed poorest learning in the continuous and discrete probabilistic tasks. We assessed the effect of a deterministic reward landscape (Fig. 1b, bottom row) on learning with the continuous and discrete targets. Fig. 6a compares the time courses for the 3–8 year olds across all four tasks. There was not a significant difference in mean age between the four tasks (*F*_3,168_ = 1.072, p = 0.362) nor was there a significant interaction between task and sex on learning ability (*F*_3,164_ = 0.97, p = 0.409). Learning was worst under the continuous probabilistic task, followed by the discrete probabilistic task and continuous deterministic task. The 3–8 year olds performed best on the discrete deterministic task and in contrast to the other three tasks, show a similar time course of learning as adults for the same task (Supp. Fig. 13). ANOVA of final learning (Fig. 6b left panel) showed a significant effect of Target type (discrete better than continuous; *F*_1,168_ = 12.87, p < 0.001) and Reward landscape (deterministic better than probabilistic; *F*_1,168_ = 43.66, p < 0.001) with no interaction (*F*_1,168_ = 0.24, p = 0.628). This shows that making the task discrete or deterministic improves learning and that these factors were additive. This was not due to significant differences in baseline variability (Fig. 6b 2nd panel; Target type: *F*_1,168_ = 0.16, p = 0.69, Reward landscape: *F*_1,168_ = 0.56, p = 0.455, interaction: *F*_1,168_ = 1.71, p = 0.192).

We examined variability in the success and fail clamp blocks as a function of Target type and Reward landscape. For the success clamp block, there was no main effect of Target type (*F*_1,168_ = 0.07, p = 0.79), but a main effect of Reward landscape (*F*_1,168_ = 6.71, p = 0.01; less variability with a deterministic landscape) with no interaction (*F*_1,168_ = 3.58, p = 0.06). This is consistent with there being no advantage to explore after success under a deterministic reward landscape, whereas there is under a probabilistic landscape (exploration can lead to locations that have higher probability of reward). For the fail clamp block there was a main effect of both Target type (*F*_1,168_ = 29.93, p < 0.001; greater variability for discrete targets) and Reward landscape (*F*_1,168_ = 9.26, p = 0.003; greater variability with a deterministic landscape) with no interaction (*F*_1,168_ = 1.01, p = 0.316). The increased variability for discrete targets is likely because participants must move to a new target to explore resulting in a larger position change. Increased variability after failure in the deterministic landscape is likely because a failure at one location predicts there will never be success at that location (in contrast to the probabilistic tasks) thereby encouraging exploration. These show that even young children choose their exploration in a rational way based on tasks features.

## Discussion

We studied a large, cross-sectional cohort of 3 to 17-year-old children and adults performing reinforcement-based motor learning tasks. Binary feedback—success or failure—was the only learning signal provided as participants moved to continuous or discrete targets under probabilistic or deterministic reward landscapes. The continuous target gave them unlimited movement choices across a target zone, whereas discrete targets constrained choices. Reward probability mapped to movement endpoint position, with the probabilistic landscape varying from 25% to a small 100% region, and the deterministic landscape with a single, small 100% reward zone. We found a developmental trajectory of reinforcement learning in the continuous probabilistic task, with older children learning more than younger children. This was paralleled by age-dependent increases in exploration after a failed trial which is essential for learning and decreases in baseline movement variability. A mechanistic model of the task revealed that in addition to the increase in exploration with age, there was a beneficial reduction in motor noise with age. The model also indicated that the learning rate increased with age. The learning rate was a proportion of the exploration (that led to success) that was incorporated into the estimate of where participants should move in future trials; higher learning rates meant that they updated more.

In contrast to the children in the continuous probabilistic group, children in the discrete targets or deterministic reward groups showed better learning. Moreover, these effects appeared to be additive—the 3to 8-year-old children learned best with discrete targets *and* in a deterministic reward landscape. Thus, the youngest children had the fundamental mechanisms for interpreting binary reward signals to drive reinforcement learning of movement—this is not surprising given that this ability has been shown in infants [3]. However, children aged 3 to 8 did not effectively utilize reward signals to learn in situations where they had to respond to probabilistic environments or where there were no spatial cues specifying distinct targets.

Our data suggest that the developmental trajectory we identified was not due to poor motor accuracy or lack of understanding of the task in the younger children. We designed the baseline block to ensure that children could accurately hit individual targets presented in different locations across the screen. The width of these targets was the same as that of the hidden 100% reward zone within the learning block and children of all ages could hit these targets accurately. The youngest children could also learn similarly to adults in the discrete deterministic task. This shows that children were able to understand the concept of the task and how to be successful.

Movement variability in our task can be due to motor noise (unintentional) as well as active exploration (intentional). While experimentally, it is challenging to separate the two sources of variability, our reinforcement learning model allows us to individually characterize motor noise and exploration. Simulations with our fit parameters approximated the observed learning behavior suggesting that these two components of variability are the drivers of learning performance. Our model suggests that with age, motor noise decreases and exploration variability increases making one more able to learn effectively within a probabilistic learning environment.

Why do discrete targets improve learning performance? In our tasks, a child needs to remember their previous endpoint position and whether it was rewarded to decide where to move at the start of the next trial. We observed that tasks with the continuous target were harder for younger participants to learn, and suspect this is because spatial working memory is not fully developed [17], particularly in those under 10 years of age [18–20]. Younger children may benefit from clear markers in the environment to differentiate various spatial positions along the target. This is consistent with tasks that have assessed children’s preferences for reporting information; they find it easier to select from discrete choices on a Likert scale versus using a continuous visual analog scale [21, 22]. Finally, it is also possible that older children had the ability to use different working memory encoding mechanisms. It is known that phonological encoding using verbal labels develops later than visual encoding and both can be used to hold information in working memory (e.g., my last movement was near the left edge of the screen) [18, 19]. Future work could explore whether providing an explicit verbal strategy to younger children could improve their ability to explore more effectively with a continuous target, highlighting the interplay of cognitive and motor domains in reinforcement learning.

Why did deterministic reward feedback improve learning? The deterministic landscape is less ambiguous than the probabilistic landscape by design—participants always fail if they are outside the 100% reward zone. The need to track and evaluate the reward frequency across target zones is eliminated, making the task less complex. Younger children have been reported to take longer to choose a reward maximization strategy in a probabilistic environment where all choices have the possibility of being rewarded. Plate et al. showed that when children and adults choose between eight options, they initially probability match (i.e., the frequency of selection closely matches the frequency of reward on the various task options). However, adults switch over to a maximizing strategy (i.e., sticking to the highest reward option) more quickly than children [23]. In a deterministic landscape, probability matching would result in the same behavior as reward maximizing and therefore the younger children’s behavior would appear nearly the same as adults. Young children’s behavior may also stem from a fundamental need to prioritize hypothesis testing and gathering information about the world [24–26], a discovery process that facilitates increased knowledge about the environment’s causal structure [27].

Our interpretation is that poorer learning performance in tasks besides discrete deterministic was due to inability to effectively utilize probabilistic reward signals or find the high reward location without clearly delineated spatial cues. This has significant ramifications as most objects in the world do not have delineated targets on them; we learn which location on an object leads to reward by exploring different options (e.g., the best location to grab a mug or push a heavy door open). The world is also not deterministic, as it is rare that the same action will always give the same result. Movement outcomes are probabilistic due to both environmental variation and motor noise (e.g., the direction of a soccer ball when kicked on different fields or the location of a thrown dart on a dartboard). Eventually, children must learn how to move successfully to interact with their environments using such probabilistic signals.

The differential ability to incorporate reward signals into changes in behavior across childhood may stem from maturation of the reward centers in the brain. Structures important for these processes, such as the basal ganglia reward centers, dorsal lateral prefrontal cortex, posterior parietal cortex, and the anterior cingulate cortex develop throughout childhood [28–30]. As a main underlying neural structure of reinforcement learning, the pallidum reaches peak volume at 9.5 years for females and 7.7 years for males while it takes the thalamus until 13.8 years for females and 17.4 years for males to reach peak volume [28]. Older children have more mature brain circuits and may be better able to take advantage of strategies in probabilistic environments that younger children cannot [24, 25]. For example, older children might know to avoid a location as soon as a failure occurs, even if that location was previously rewarded. Younger children might continue sampling those locations, perhaps due to immature signaling in the brain. Indeed, it has been shown that brain activity in younger children can be similar after positive and negative rewards whereas in older children and adults it is more distinct [31–33].

Online studies such as ours inevitably have some limitations. Given that this task was conducted remotely, we did not have control of the computer operating quality, internet speed, or testing environment for our participants (see Methods for exclusion criteria). As such, we were not able to control or analyze timing parameters of movement which can be done more easily in in-person experimentation. However, our key analyses compare across large groups which likely factor out these uncontrolled variables. Our results (comparison of in lab vs. online and fit of a noise model vs. full reinforcement learning model) also confirm that even the youngest age group understood the task.

Our findings in typical development lay a foundation for better understanding of behavior during childhood and could help inform what function may be lost or impaired after injury. This underscores the need to consider not only disease process for interventions but also age as there are developmental differences in motor learning capacity in individuals at different ages. Knowing how the sensorimotor system works at different ages can guide decisions on how to intervene or alter an environment and give children the best opportunity to use reinforcement learning mechanisms for rehabilitation outcomes.

## Methods

### Participants

Children and adults without neurological impairment or developmental delay were recruited to one of four experiments as outlined in Table 1. A total of 385 complete datasets were included in the analysis. The continuous probabilistic task was designed first and the other three tasks, discrete probabilistic, continuous deterministic, and discrete deterministic, were conducted to expand upon the results of the first task and further identify factors contributing to learning by reinforcement across the developmental spectrum. Participants were recruited from the Johns Hopkins University community through an online announcement portal, the greater Baltimore Maryland area through Research Match, and nationwide through the online platform Lookit which merged with Children Helping Science in 2023 [34, 35]. Our sample includes participants from 38 states out of the 50 United States of America (Supp. Fig. 1). Each participant was screened to rule out neurological impairment, developmental delay, and other developmental disorders that could affect motor and cognitive development. This study was approved by the Johns Hopkins School of Medicine Institutional Review board and all participants, or their parent/legal guardian, provided informed consent prior to participation.

### Task Platform

All four tasks were completed on a web-based platform built with Javascript as previously reported by [36] with modifications to the game environment and feedback type. This platform allowed creativity in the game environment design to be kid friendly and engaging to young participants to foster sustained attention and increase likelihood of completing the full task. The task platform allowed remote completion of the experiment by participants on their home computer or tablet. A small subset of participants in the continuous probabilistic task (n = 16; 3 to 5yo n = 2, 6 to 8yo n = 3, 9 to 11yo n = 5, 12 to 14yo n = 5, 15 to 17yo n = 1) completed the task in person in the research laboratory and the remainder of participants completed the task remotely. Participants used a mouse, trackpad, or touchscreen input to control a cartoon penguin game piece and move across the game environment. Movement trajectories were sampled at the polling rate of the selected input device and data from each trial were uploaded and saved to Google Firebase Realtime Database at the end of each trial. Due to the remote data collection nature of this experiment, we were not able to control data sampling rates. Each trial was manually inspected and sampling rates of input devices had a mean of 37.363 ± 4.184 Hz.

### Procedure

The game environment is an immersive icy landscape with penguins. The overall goal of the task was to move a penguin from the starting position on one side of the ice (at the bottom of the computer or tablet screen closest to the participant) to a distance of 24 game units (GU) into the game (at the far edge of the screen away from the participant). If successful, a pleasant sound would play, the video screen above the ice would be outlined in blue, and a Disney video clip (different on each trial; gifs hosted on https://giphy.com) would play. Multiple signals of reward were provided to ensure that participants associated their behavior with the feedback provided and could clearly differentiate between a successful trial and a failure trial. The penguin game piece started as a blue color to indicate that it was not active. To activate the penguin, the participant had to click the left mouse button (mouse or trackpad) or touch and hold the penguin and it would turn white to indicate that it was movable. Then the participant made a reaching movement to move the penguin across the ice. The trial would end when the Y position of the penguin exceeded 24 GU. To account for variability in input device sampling rates, the final X position was computed as an interpolation between the data points immediately before and after Y = 24 GU such that every trial had a calculated X position at Y = 24 GU.

Rewards were determined based upon the interpolated final X position of the game piece at the back edge of the ice and a task specific reward landscape. All tasks included five blocks as outlined in Fig. 2a. Baseline (20 trials): a single, discrete target (image of three dancing penguins) was presented at a randomized X position at the far edge of the ice. Participants were instructed to move accurately to the target. Learning (100 trials): participants were exposed to one of four learning environments with variations in the target type (continuous vs. discrete, Fig. 1a) and the reward feedback (probabilistic vs. deterministic, Fig. 1b). In the continuous conditions, participants could choose to move the penguin to any location on a continuous horizontal target (Fig. 1a – continuous). In the discrete conditions, participants could move to one of seven targets spread horizontally (Fig. 1a – discrete). For probabilistic feedback, the reward was determined by an unseen position-based probability gradient with a small 100% reward zone away from which reward probabilities decreased linearly to a baseline (Fig. 1b – continuous probabilistic and discrete probabilistic). For deterministic feedback, reward was always given within a reward zone but not elsewhere (Fig. 1b – continuous deterministic and discrete deterministic). Success Clamp (10 trials): every trial in this block was rewarded regardless of the final X position of the game piece. Fail Clamp (10 trials): no trials in this block were rewarded. Single Target (10 trials): the same single, discrete target as in baseline was presented in the center of the far edge of the ice and participants were cued to move accurately towards it. A break for a new set of instructions was provided between Baseline and Learning as well as Fail Clamp and Single Target. Participants were unaware of the reward criteria transitions between Learning and Success Clamp and Success Clamp and Fail Clamp. The ideal/mature behavior in these tasks was to explore at the beginning of the learning block to find the area of most frequent reward and then exploit this behavior to maximize reward for the remaining trials of the block. Moreover, if the 100% reward zone has been found successfully, continuing to move to this reinforced position during the success clamp and then exploring again in the fail clamp are indicators of mature sensitivity to reward and failure.

### Continuous Probabilistic

In the Learning block for the continuous probabilistic task, participants were presented with a continuous target and probabilistic reward feedback (Fig. 1b – continuous probabilistic). The probability reward landscape is defined by setting reward percentage probabilities at 5 specific x locations with reward between these locations being linearly interpolated (and constant outside the range). We set X values of {−24, −14.5, −9.5, 1.1875} to reward probabilities {33, 100, 100, 25}.

Participants were warned that some of the ice was starting to melt which would cause the penguin to slip and were told that in some places they would slip a lot of the time, some places they would slip some of the time, and in some places they would never slip. They were instructed to move the penguin across the ice without slipping to get the movie clip to play as often as possible. If they were not successful, the penguin would fall over, and they would see a sad face penguin image before the game reset for the next trial Fig. 1c. This task design builds from Cashaback et al. where participants were asked to reach to any location on a continuous line, with the probability of being rewarded dependent on the reach end location. In their task, there was a small (hidden) 100% reward zone with reward probability decreasing on either side away from this zone. They found that adult participants could learn from a probabilistic reward landscape and find the rewarding zone [13]. We explored a similar task design in participants across development.

### Discrete Probabilistic

In the Learning block for the discrete probabilistic task, participants were presented with a set of targets, each associated with a specific probability of reward (Fig. 1b – discrete probabilistic). We set the center of seven the targets at X values of {−18, −12, −6, 0, 6, 12, 18} with target width of 5 and with reward percentage probabilities of the {66, 100, 66, 33, 25, 25, 25} (Fig. 1b bottom left).

The discrete targets were visually the same as those used in the Baseline and Single Target blocks, however all seven were presented at the same time in equally spaced positions across the edge of the ice. Participants were instructed to find the group of penguins that made the video clip play all the time by moving their penguin game piece across the ice. They were told to move to one group of penguins on each trial and that some penguins would make the movie clip play some of the time but there was a group of penguins that played the clip all the time. If they were not successful, they would see a red X on the video screen and the video clip would not play before the game reset for the next trial. To ensure that participants could accurately distinguish the feedback associated with each target, there was a visible space between each target. If the participant moved between targets, the participant would receive a message to try again, and the trial would reset until one target was accurately hit.

### Continuous Deterministic

In the Learning block for the continuous deterministic task, participants were presented with a continuous target and deterministic reward feedback (Fig. 1b – continuous deterministic). Participants were warned that some of the ice was starting to melt which would cause the penguin to slip and were told that in some places they would slip all of the time and in some places they would never slip. They were instructed to move the penguin across the ice without slipping to get the movie clip to play as often as possible. If they were not successful, the penguin would fall over, and they would see a sad face penguin image before the game reset for the next trial. This task was completed by a subset of participants aged three to eight years and adults.

### Discrete Deterministic

In the Learning block for the discrete deterministic task, participants were presented with a set of seven discrete targets and deterministic reward feedback (Fig. 1b – discrete deterministic). Participants were instructed to move across the ice to one of the groups of penguins on each trial to get the movie clip to play. They were told that one group of penguins would make the video clip play all of the time. If they were not successful, they would see a red X on the video screen and the video clip would not play before the game reset for the next trial. As in the discrete probabilistic task, to ensure that participants could accurately distinguish the feedback associated with each target, there was a space between each target. If the participant moved between targets, the trial would reset until one target was accurately hit. This task was completed by a subset of participants aged three to eight years and adults.

### Demo Versions of Tasks

Shortened versions of each task without data collection are provided at the following links. In these versions, there are 5 trials in baseline, 10 trials in learning, 2 trials in each clamp, and 3 trials of single target. To proceed beyond the information input screen, use an arbitrary 6-digit code for the subjectID and participant information to sample the game environment.

Continuous Probabilistic: https://kidmotorlearning.github.io/PenguinsDemo_Continuous-Probabilistic/

Discrete Probabilistic: https://kidmotorlearning.github.io/PenguinsDemo_Discrete-Probabilistic/

Continuous Deterministic: https://kidmotorlearning.github.io/PenguinsDemo_Continuous-Deterministic/

Discrete Deterministic: https://kidmotorlearning.github.io/PenguinsDemo_Discrete-Deterministic/

### Measurements and Analysis

We used several metrics to analyze performance and variability in different blocks and evaluate reinforcement learning over childhood. Baseline performance was defined as the distance from the target center averaged over each of the 20 baseline trials. Baseline variability was defined as the standard deviation of the baseline performance. Learning performance (*Distance*) is defined as the absolute value of the interpolated X position distance from the center of 100% reward zone (located at X = −12 in all tasks) averaged over the last 10 trials of the learning block. A *Distance* value closer to 0 indicates better learning. To quantify variability after success, we used the standard deviation of the interpolated X position in trials 2 through 10 of the success clamp block. To quantify variability after failure, we used the standard deviation of the interpolated X position in trials 2 through 10 of the fail clamp block.

Participant characteristics (sex and game play handedness) and game play parameters (device and browser) were analyzed in one way ANOVAs with dependent variable of *Distance* to determine whether these parameters influenced learning ability. To determine whether there was a differential effect of sex, device, or handedness on learning performance in the four different tasks, additional two way ANOVAs with dependent variable of *Distance* were used. There was not a significant interaction between task and sex (reported in Results), task and device (*F*_3,168_ = 0.62, p = 0.717), or task and handedness (*F*_3,168_ = 1.2, p = 0.312). Each trial was divided into three phases to describe the reaction, stationary, and movement times. Reaction time is defined as the time from the appearance of the penguin (start of the trial) to the time the penguin is clicked and activated. Stationary time is defined as the time from penguin click to movement onset (first non-zero position). Movement time is defined as movement onset to end of the trial when the penguin moves across the back edge of the ice. Trial timings for each phase of movement were extracted and averaged for each participant across the whole experiment and then averaged within individual age bins to evaluate across ages. Total game play time was also extracted and averaged by age bins. Path length ratios were calculated as the actual path length from the start to end of the trajectory divided by the ideal straight-line path from the first position in the trajectory to the last position in the trajectory. The path length ratio for all trials were averaged for each participant and then averaged within age bins for comparison between ages.

We used linear regression, mediation analysis, and one and two-way ANOVAs to evaluate effects of age and other outcome variables on learning as well as compare performance between tasks. Significance level of 0.05 was used for all statistical tests. All raw data and statistical analyses were completed using custom scripts in MATLAB (version R2023a). We regressed Age with each performance or variability metric independently as well as in a multiple linear regression model. To compare performance by age bin for the children (5 bins of 3 years each spanning age 3 to 17 years) between the baseline block of discrete targets and the final block of discrete targets, we used a repeated measures ANOVA with a grouping variable of age bin. We used a mediation analysis model with Age as the independent variable (X) and *Distance* as the dependent variable (Y). For the continuous probabilistic task, the mediators (M1 and M2) were Baseline Variability and Variability after Failure. For the discrete probabilistic task, the mediators (M1 and M2) were Variability after Success and Variability after Failure. For each model, the five path coefficients were calculated describing the relationship of X to M (path *a*), M to Y (path *b*), X to Y (path *c*), X to Y mediated by M (path *c*′) and the mediation effect (path *ab*). Mediation analyses were completed using a MATLAB Mediation Toolbox (https://github.com/canlab/MediationToolbox; original access date June 1, 2023).

To investigate the influence of a previous failure on selection of the next movement, we used a regression analysis. Equation 1 describes the relationship between change in position from the immediately previous trial weighted by experiencing a failure on up to three recent trials.


(1) $$\left|\Delta x_{t}\right|=w_{0}+w_{1}F_{t-1}+w_{2}F_{t-2}+w_{3}F_{t-3}$$

where:

$\Delta x_{t}=x_{t}-x_{t-1}$; change in position from the previous trial

$w_{0}$= inherent exploration if no failures on previous 3 trials

$w_{i}$= weight of failure on the t-i^*th*^ trial

$F_{t-i}$= failure status of the t-i^*th*^ trial; 1 = fail, 0 = success

### Reinforcement learning model

To account for the reaching behavior, we developed a probabilistic model that incorporates both exploration variability and motor noise. The data consists of the observed reach locations, $s_{t}$, on trial t and the reward $r_{t}$ (1 or 0 depending on whether the reach was rewarded or not). Our model builds from Therrien et al. who examined how participants used binary deterministic reinforcement of reaching movements to find a rewarded endpoint location that moved over time. Using a mechanistic model, they showed that the ability to learn depended upon appropriate exploration variability relative to their motor noise (i.e., variability that is not under the control of the subject) [14].

We assume that on each trial the participant has an internal estimate of the desired reach location, $x_{t}$, which they can update as the experiment proceeds. On each trial we include variability in a participant’s produced reach location from two potential sources – motor noise $\left(m_{t}\right)$ and exploration variability $\left(e_{t}\right)$. The key difference between these two sources of variability is that we assume participants can use the exploration variability, but not their motor noise, to update their desired reach location. On each trial, each source of variability is modeled as a draw from a zero-mean Gaussian distribution. For motor noise the standard deviation of the Gaussian is $\sigma_{m}$ which is constant across trials. We assume that subjects only explore after a unrewarded trial and that the exploration variability is a draw from a zero-mean Gaussian distribution with standard deviation $\sigma_{e}$.


(2) $$e_{t}\sim 𝓝\left(0,\left(1-r_{t-1}\right)\sigma_{e}\right)$$

The produced reach, $s_{t}$, is then the desired reach with the addition of the variability from motor noise and exploration (if any), given by the *output* equation:

(3) $$s_{t}=x_{t}+\left(1-r_{t-1}\right)e_{t}+m_{t}$$

The probability p of reward received, $r_{t}$, depends on the the actual reach location $s_{t}$ and the particular reward regime used such that the *reward* equation is

(4) $$p\left(r_{t}=1\right)=f\left(s_{t}\right)$$

where f() can represent different functions such as the continuous or discrete probabilistic reward regimes.

After each reach, the participant updates their desired reach location only if they were successful. They update the reach to incorporate part of any exploration so that the *update* equation is

(5) $$x_{t+1}=x_{t}+\eta r_{t}e_{t}$$

where η is a learning rate between 0 and 1.

#### Model fitting

To fit this stochastic model to each participant’s data, we used a Kalman filter. The model has 3 parameters:$\theta =\left\{\sigma_{m},\sigma_{e},\eta \right\}$. We can reformulate the equations above into a standard Kalman filter. The update equation is given by

(6) $$\left[\begin{array}{c} x_{t}\\ e^{t} \end{array}\right]=\left[\begin{array}{cc} 1 & \eta r_{t-1}\\ 0 & 0 \end{array}\right]\left[\begin{array}{c} x^{t-1}\\ e^{t-1} \end{array}\right]+\left[\begin{array}{c} 0\\ \left(1-r_{t-1}\right)\sigma_{e} \end{array}\right]v_{t}$$

where $v_{t}\sim 𝓝\left(0,1\right)$. The output equation is given by

(7) $$s^{t}=\left[\begin{array}{cc} 1 & 1 \end{array}\right]\left[\begin{array}{c} x^{t}\\ e^{t} \end{array}\right]+\left[\sigma_{m}\right]w_{t}$$

where $w_{t}\sim 𝓝\left(0,1\right)$. We initialized the desired state as the median of the first five reach locations and the state covariance as

(8) $$\left[\begin{array}{cc} 1 & 0\\ 0 & \sigma_{e}^{2} \end{array}\right]$$

We used Matlab fminsearchbnd to fit the parameters to maximize the likelihood over the 100 learning trials for each participant. This ensured that our parameter fits were not contaminated by behavior in the clamp block and also allowed us to try and predict the out-of-sample clamp behavior. In addition, we also examined parameter recovery by generating simulated data (for each participant using their best fit parameters) and comparing the fit values with the true values used to simulate the data. We also fit data from the discrete probabilistic task in the same way as the continuous (so that the model does not know about the discrete nature of the task).

## Supplementary Material

Supplement 1

## Figures and Tables

**Table 1.** Participant Demographics Ethnicity and race classifications were self-reported by participant/parent. Participants who identified as two or more categories of race (Black, White, and/or Asian) were classified as Multiple. Participants who specified Asian (Indian) or South Asian were classified as Asian. Participants who identified as one or more races other than Black, White, or Asian were classified as *Other*.

	Kids				Adults			
Task	Cont. Prob.	Dis. Prob	Cont. Det.	Dis. Det.	Cont. Prob.	Dis. Prob	Cont. Det.	Dis. Det.
*n*	111	106	40	41	33	33	10	11
*% Female*	41	57	38	39	67	58	60	82
*% RH*	95	96	93	78	97	97	100	91
Age (yrs)								
*Mean (std)*	9.98 (4.2)	9.46 (4.1)	5.4 (1.7)	5.44 (1.5)	25.18 (4.6)	24.42 (3.9)	24.9 (3.7)	25.9 (4.5)
*Median*	10	10	5.5	5	24	24	25	27
*Range*	3–17	3–17	3–8	3–8	18–35	18–31	20–32	18–34
N per age bin								
*3–5yrs*	21	22	20	21				
*6–8yrs*	27	21	20	20				
*9–11yrs*	20	28						
*12–14yrs*	20	19						
*15–17yrs*	23	16						
Device								
*Mouse*	69	61	15	19	17	22	7	8
*Trackpad*	16	38	12	14	15	11	3	0
*Touchscreen*	26	7	13	8	1	0	0	3
		Ethnicity		Race				
		Hispanic	Not Hispanic	Black	White	Asian	Multiple	Other

Kids (n,%)		19, 6.38	279, 93.62	15, 5.03	234, 78.52	17, 5.71	24, 8.05	8, 2.68
Adults (n,%)		3, 3.45	84, 96.55	6, 6.90	34, 39.08	41, 47.12	5, 5.75	1, 1.15

Abbreviations: Cont., continuous; Det., deterministic; Dis., discrete; n, number; Prob, probabilistic; RH, right-handed; std, standard deviation; yrs, years.

## Data Availability

Data are available upon reasonable request to the corresponding author.
