# Supplementary material for: Age-dependent predictors of effective reinforcement motor learning across childhood
Source: bioRxiv. 2025 Mar 15:2024.07.09.602665. Originally published 2024 Jul 9. Preprint. [Version 2] doi: 10.1101/2024.07.09.602665 (PMC11257637; doi:10.1101/2024.07.09.602665)
Supplement: Supplement 1 [file NIHPP2024.07.09.602665v2-supplement-1.pdf]

bioRxiv preprint doi: https://doi.org/10.1101/2024.07.09.602665; this version posted March 15, 2025. The copyright holder for this preprint (which was not certified by peer review) is the author/funder, who has granted bioRxiv a license to display the preprint in perpetuity. It is made available under aCC-BY 4.0 International license.

953 # Supplementary Information

Task	Measure	Factor	F	p
**Continuous Probabilistic**				
	Baseline Precision	Sex	$F_{1,142} = 0.251$	p = 0.617
		Hand	$F_{1,142} = 0.213$	p = 0.645
		Device	$F_{2,141} = 1.066$	p = 0.347
		Browser	$F_{3,140} = 0.414$	p = 0.743
	Distance from Target	Sex	$F_{1,142} = 0.740$	p = 0.391
		Hand	$F_{1,142} = 0.815$	p = 0.368
		Device	$F_{2,141} = 0.733$	p = 0.482
		Browser	$F_{3,140} = 0.313$	p = 0.816
**Discrete Probabilistic**				
	Baseline Precision	Sex	$F_{1,137} = 0.431$	p = 0.513
		Hand	$F_{1,137} = 0.923$	p = 0.338
		Device	$F_{2,136} = 0.677$	p = 0.510
		Browser	$F_{3,135} = 0.192$	p = 0.901
	Distance from Target	Sex	$F_{1,137} = 0.096$	p = 0.758
		Hand	$F_{1,137} = 0.645$	p = 0.423
		Device	$F_{2,136} = 1.216$	p = 0.300
		Browser	$F_{3,135} = 0.998$	p = 0.396
**Continuous Deterministic**				
	Baseline Precision	Sex	$F_{1,48} = 1.772$	p = 0.189
		Hand	$F_{1,48} = 0.544$	p = 0.464
		Device	$F_{2,47} = 0.337$	p = 0.716
		Browser	$F_{3,46} = 0.162$	p = 0.922
	Distance from Target	Sex	$F_{1,48} = 0.455$	p = 0.503
		Hand	$F_{1,48} = 0.770$	p = 0.385
		Device	$F_{2,47} = 1.482$	p = 0.238
		Browser	$F_{3,46} = 0.343$	p = 0.794
**Discrete Deterministic**				
	Baseline Precision	Sex	$F_{1,50} = 0.076$	p = 0.783
		Hand	$F_{1,50} = 0.015$	p = 0.902
		Device	$F_{2,49} = 0.691$	p = 0.506
		Browser	$F_{2,49} = 0.154$	p = 0.857
	Distance from Target	Sex	$F_{1,50} = 2.002$	p = 0.163
		Hand	$F_{1,50} = 0.105$	p = 0.747
		Device	$F_{2,49} = 0.933$	p = 0.400
		Browser	$F_{2,49} = 0.318$	p = 0.729

Supp. Tab. 1. Statistical analysis of sex, handedness, device, and browser on behavior. Results from one-way ANOVAs of participant specific factors on precision from experimental block one (baseline) and distance from target from experimental block two (learning) for each of the four tasks. For all tasks, participant specific factors did not significantly affect behavior.
