# Supplementary material for: Age-dependent predictors of effective reinforcement motor learning across childhood
Source: bioRxiv. 2024 Jul 9:2024.07.09.602665. Preprint. [Version 1] doi: 10.1101/2024.07.09.602665 (PMC11257637; doi:10.1101/2024.07.09.602665)
Supplement: Supplementary file 1 [file NIHPP2024.07.09.602665v1-supplement-1.pdf]

bioRxiv preprint doi: https://doi.org/10.1101/2024.07.09.602665; this version posted July 9, 2024. The copyright holder for this preprint (which was not certified by peer review) is the author/funder, who has granted bioRxiv a license to display the preprint in perpetuity. It is made available under aCC-BY 4.0 International license.

677 # Supplementary Information

	β	SE	Z	p
Age				
c'	-0.0225	0.0092	-2.5863	**0.0097**
c	-0.0542	0.0090	-4.0564	**< 0.001**
Baseline variability				
a_1	-0.0145	0.0038	-3.0813	**0.0021**
b_1	0.7237	0.2227	2.8329	**0.0046**
$a_1 \times b_1$	-0.0105	0.0036	-3.0341	**0.0024**
Variability after failure				
a_2	0.0363	0.0081	3.4783	**< 0.001**
b_2	-0.5851	0.0822	-3.7089	**< 0.001**
$a_2 \times b_2$	-0.0212	0.0048	-3.7021	**< 0.001**

Supp. Tab. 1. Mediation analysis for continuous probabilistic task. Results of the effect of age on learning mediated by baseline variability and variability after failure. Baseline variability and variability after failure together partially mediate the effect of age on learning. Significant effects are in bold. β: regression coefficient, SE: standard error

	β	SE	Z	p
Age				
c'	-0.0018	0.0084	-0.0883	0.9297
c	-0.0296	0.0108	-2.6794	**0.0074**
Variability after success				
a_1	-0.0169	0.0078	-2.2768	**0.0228**
b_1	0.8829	0.1136	3.3703	**< 0.001**
$a_1 \times b_1$	-0.0149	0.0069	-2.2444	**0.0248**
Variability after failure				
a_2	0.0234	0.0047	3.7185	**< 0.001**
b_2	-0.5517	0.1707	-3.3707	**< 0.001**
$a_2 \times b_2$	-0.0129	0.0046	-2.9800	**0.0029**

Supp. Tab. 2. Mediation analysis for discrete probabilistic task. Results of the effect of age on learning mediated by variability after success and after failure. Together, variability after success and after failure completely mediate the effect of age on learning. Significant effects are in bold. β: regression coefficient, SE: standard error

bioRxiv preprint doi: https://doi.org/10.1101/2024.07.09.602665; this version posted July 9, 2024. The copyright holder for this preprint (which was not certified by peer review) is the author/funder, who has granted bioRxiv a license to display the preprint in perpetuity. It is made available under aCC-BY 4.0 International license.

678 # Supplementary figures

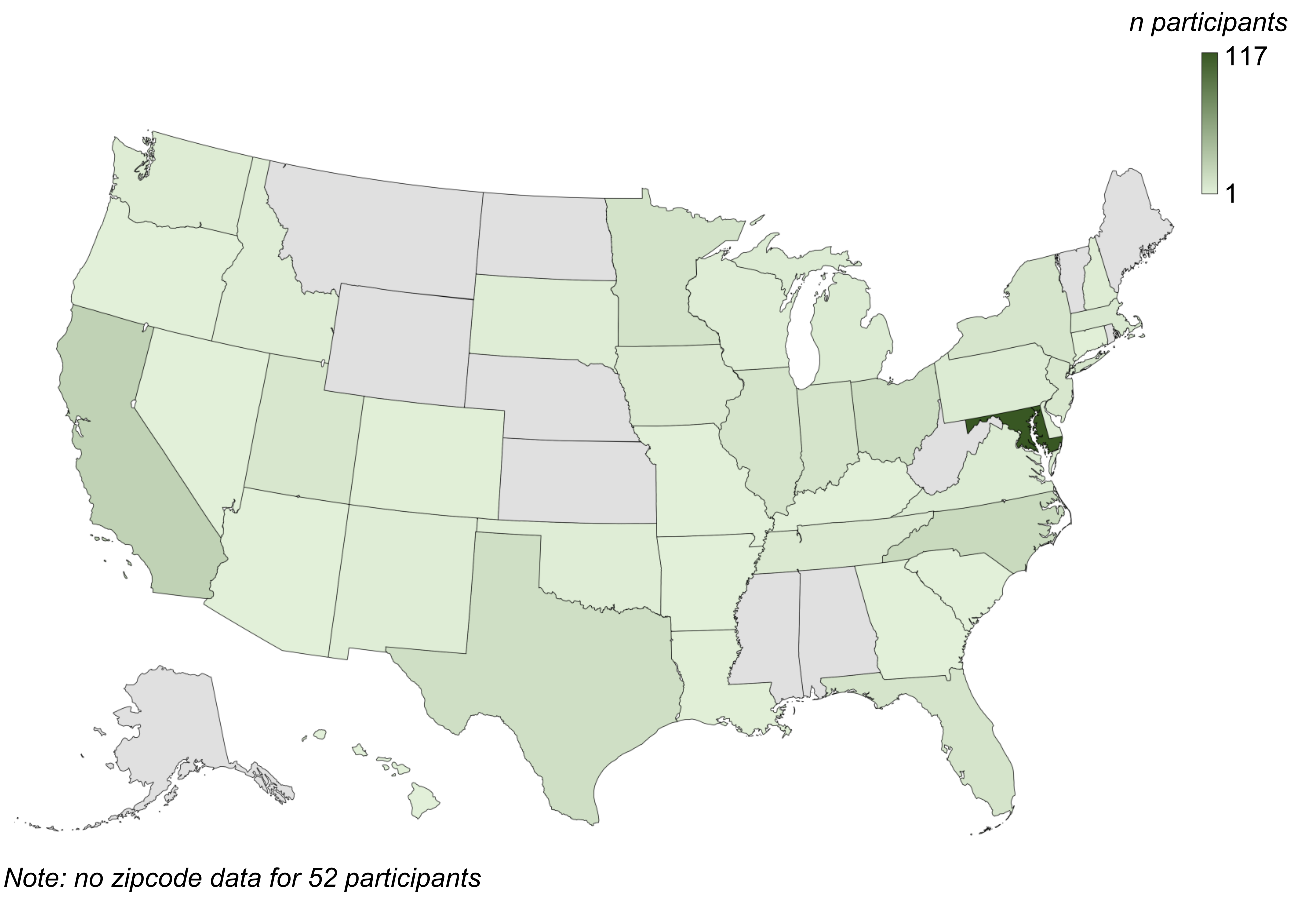

Supp. Fig. 1. Map of participant locations. Thirty-eight states of the United States of America are represented in this dataset. The map was generated in Excel on Microsoft 365.

bioRxiv preprint doi: https://doi.org/10.1101/2024.07.09.602665; this version posted July 9, 2024. The copyright holder for this preprint (which was not certified by peer review) is the author/funder, who has granted bioRxiv a license to display the preprint in perpetuity. It is made available under aCC-BY 4.0 International license.

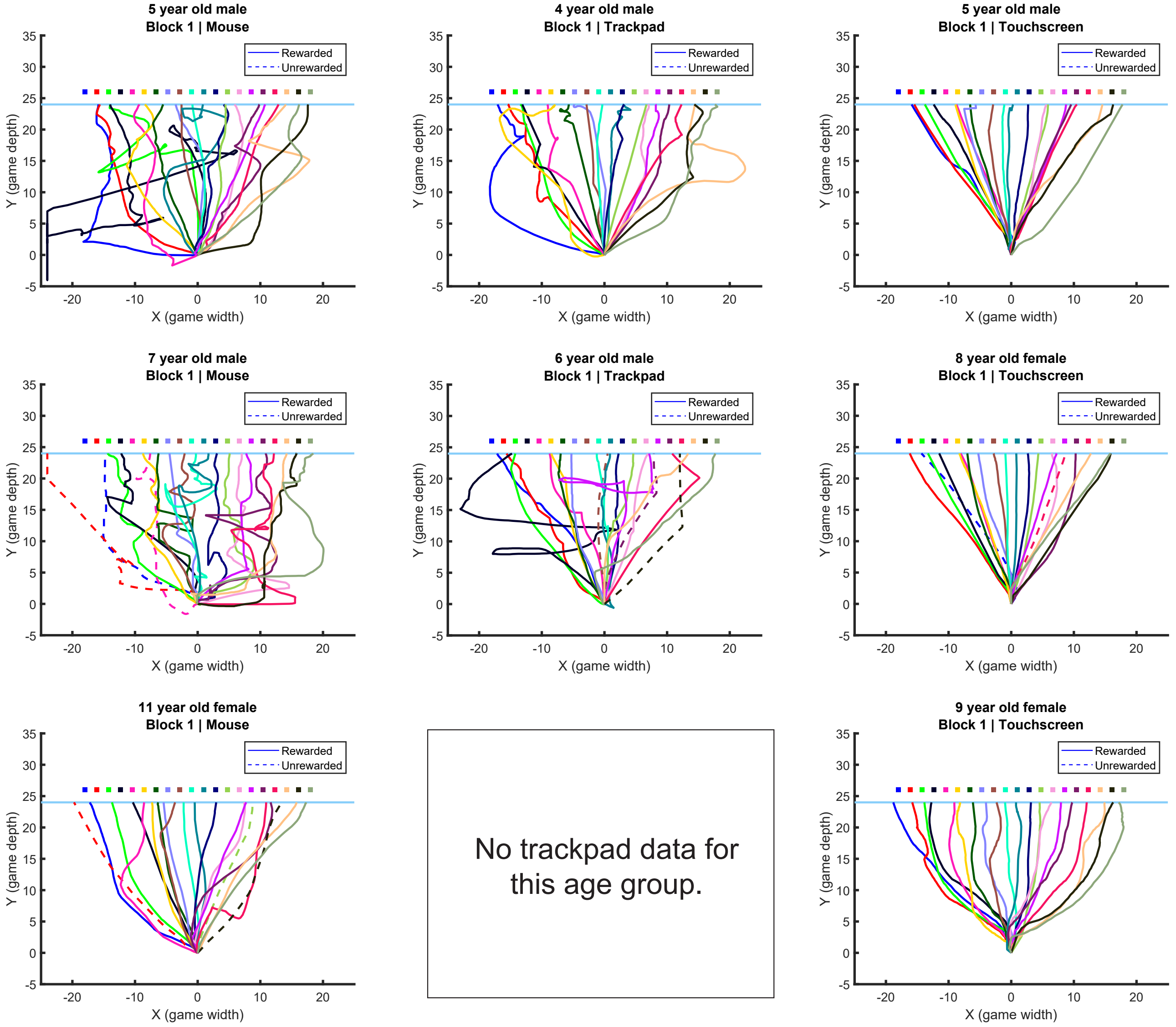

Supp. Fig. 2. Example baseline paths for participants ages 3 to 11 years old. Each trajectory begins at (0,0) and ends at Y = 24 when the penguin crosses the back edge of the ice. The final X position of each trajectory corresponds to the interpolated final position of the movement (see Methods for additional details). As available, a sample for each age bin from each input device type is provided. Note that trajectories tend to be straighter for touchscreen input compared to other devices. The twenty squares represent the target centers. Note that the full reward zone is not shown due to overlap between targets. Unrewarded and reward paths are shown as dashed and solid lines, respectively.

bioRxiv preprint doi: https://doi.org/10.1101/2024.07.09.602665; this version posted July 9, 2024. The copyright holder for this preprint (which was not certified by peer review) is the author/funder, who has granted bioRxiv a license to display the preprint in perpetuity. It is made available under aCC-BY 4.0 International license.

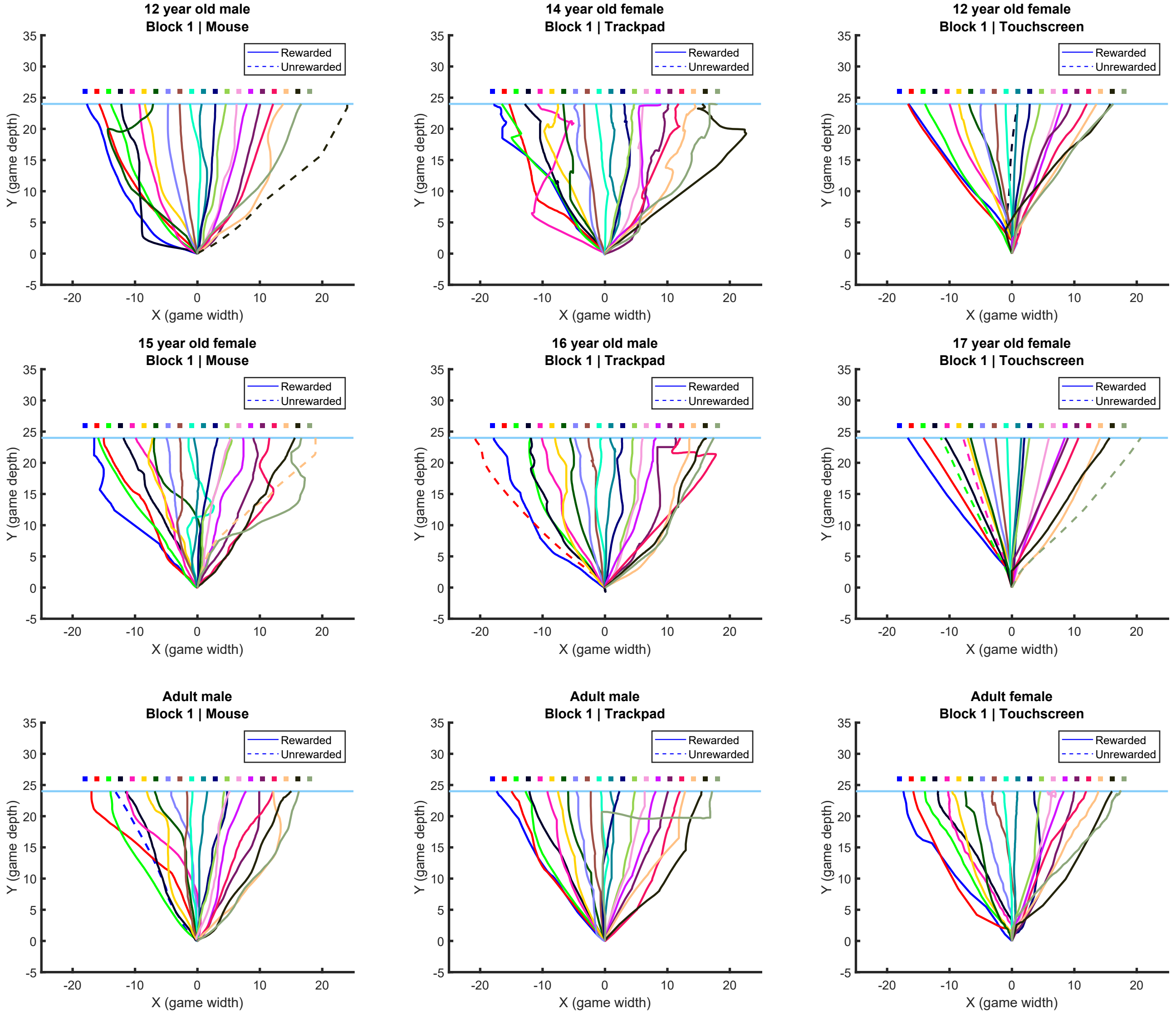

Supp. Fig. 3. Example baseline paths for participants for participants ages 12 to 17 years old and adults. Same format as Supp. Fig. 2.

bioRxiv preprint doi: https://doi.org/10.1101/2024.07.09.602665; this version posted July 9, 2024. The copyright holder for this preprint (which was not certified by peer review) is the author/funder, who has granted bioRxiv a license to display the preprint in perpetuity. It is made available under aCC-BY 4.0 International license.

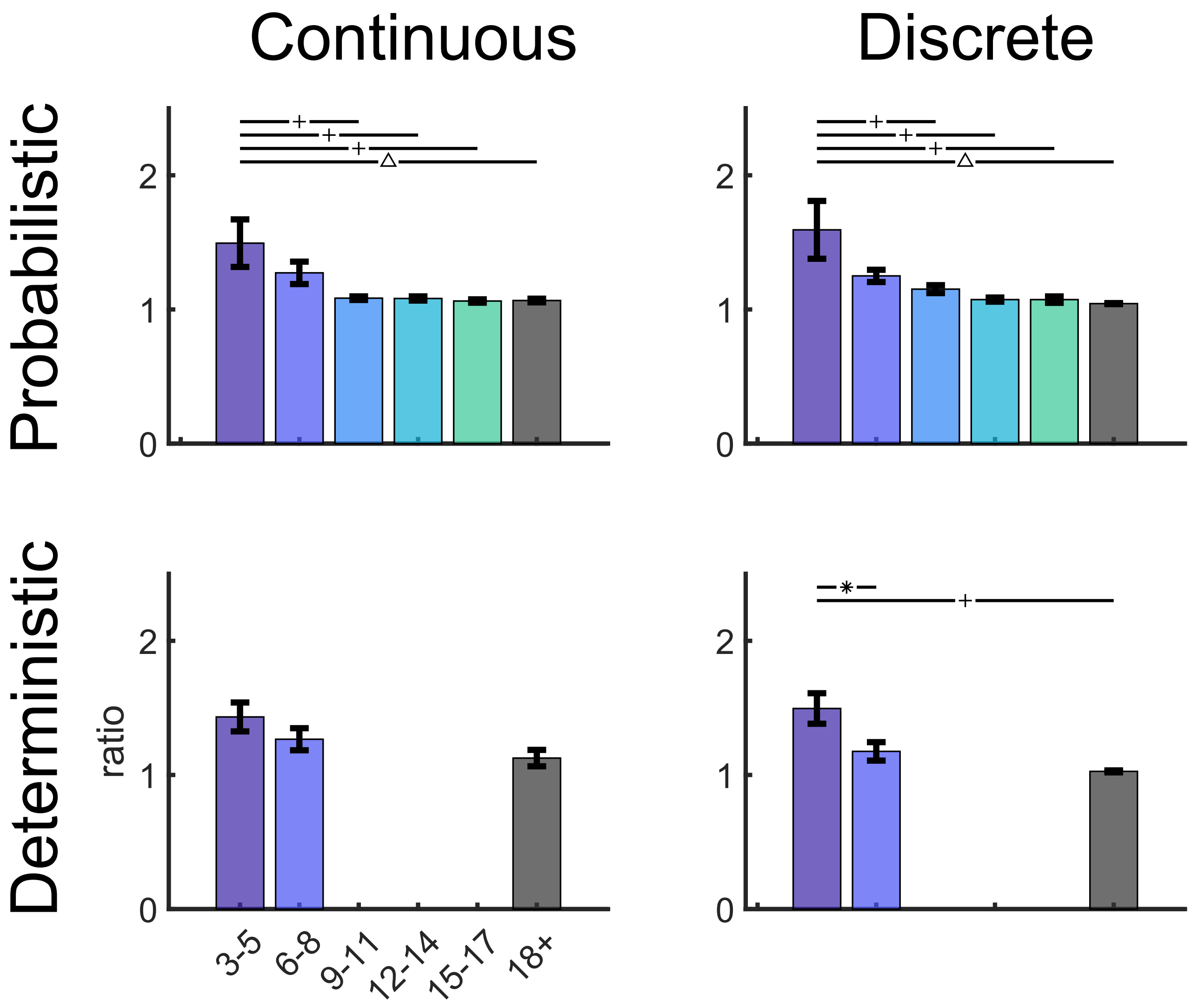

Supp. Fig. 4. Path length ratios. The path length ratio is a measure of path curvature (path length divided by distance from first to last point of movement) for the four tasks. Significant pairwise comparisons between age bins indicated above plots as follow: $*$ = $p < 0.05$, + = $p < 0.01$, and $\triangle$ = $p < 0.001$. Bars show mean and standard error of the mean.

bioRxiv preprint doi: https://doi.org/10.1101/2024.07.09.602665; this version posted July 9, 2024. The copyright holder for this preprint (which was not certified by peer review) is the author/funder, who has granted bioRxiv a license to display the preprint in perpetuity. It is made available under aCC-BY 4.0 International license.

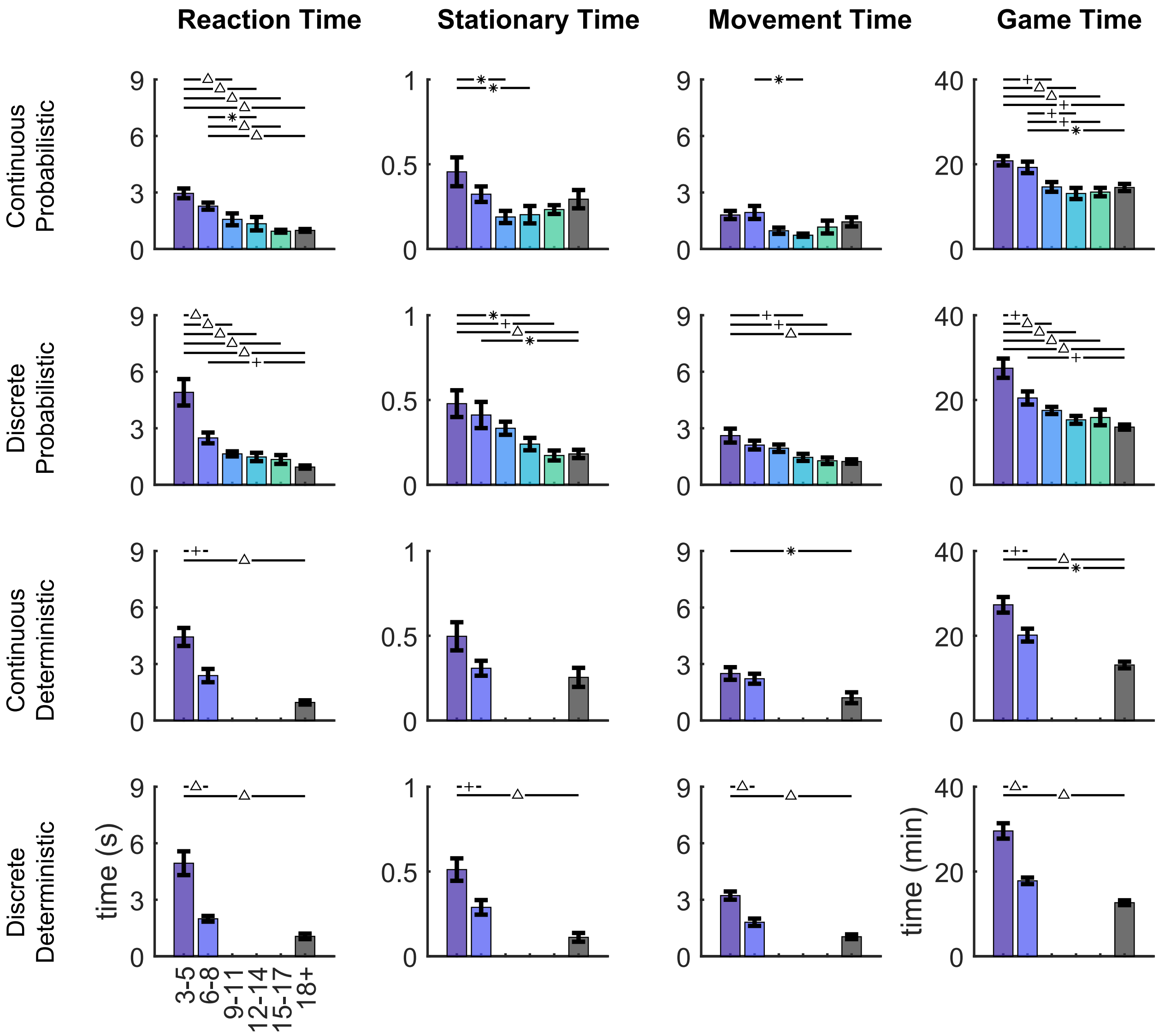

Supp. Fig. 5. Timing information. Reaction time (time from when penguin appeared until the participant clicked on the penguin to start the trial), stationary time (time from click to start of movement), movement time (time from start to end of movement) and game time (time to complete the whole task in minutes) for the 4 tasks split by age bins. Significant pairwise comparisons between age bins indicated above plots as follow: $*$ p < 0.05, + p < 0.01, Δ p < 0.001. Bars show mean and standard error of the mean.

bioRxiv preprint doi: https://doi.org/10.1101/2024.07.09.602665; this version posted July 9, 2024. The copyright holder for this preprint (which was not certified by peer review) is the author/funder, who has granted bioRxiv a license to display the preprint in perpetuity. It is made available under aCC-BY 4.0 International license.

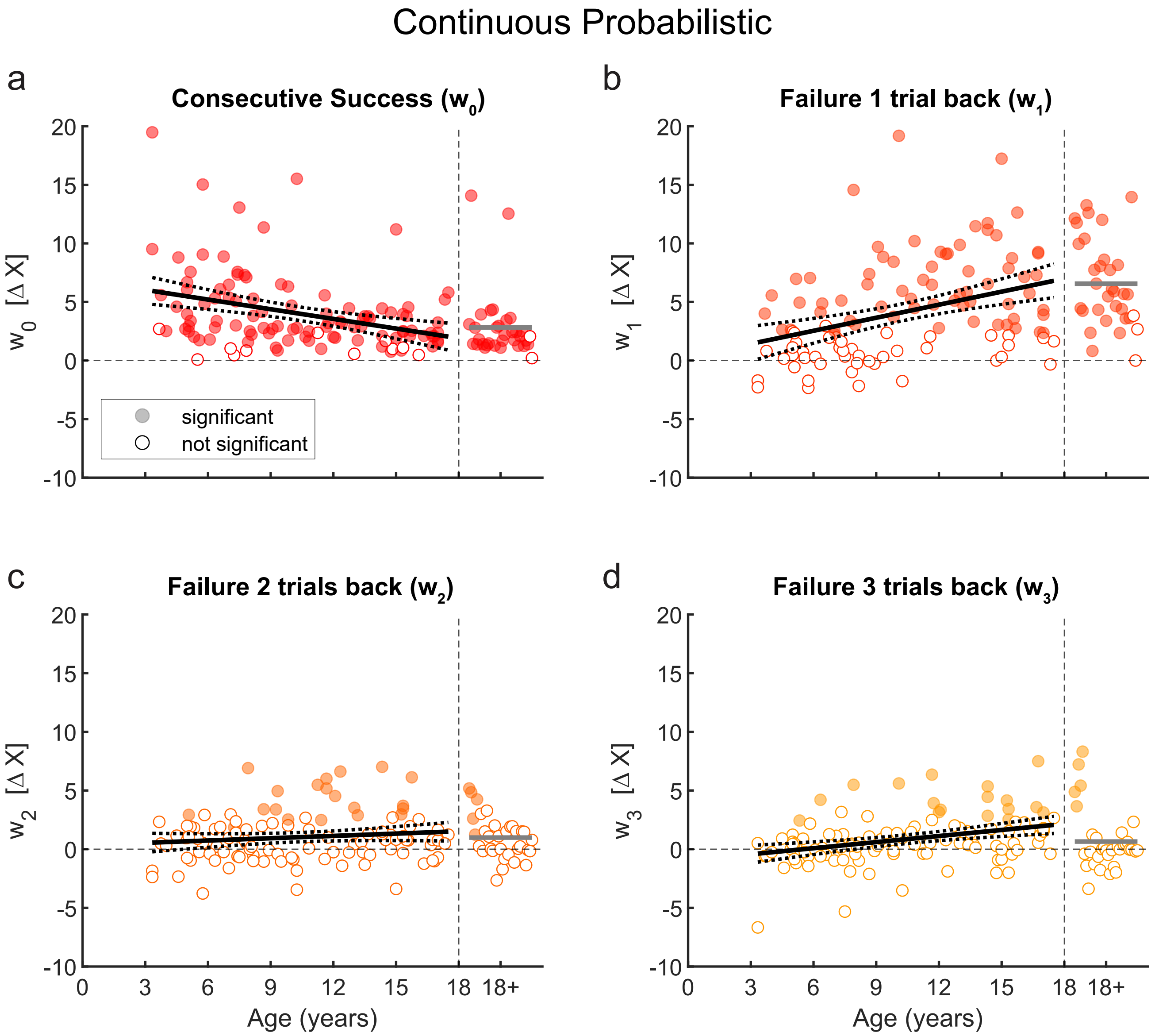

Supp. Fig. 6. History dependence of change in reach as a function of reward history for the continuous probabilistic task. We performed a regression analysis on the change in absolute reach direction ($\Delta X_t = X_t - X_{t-1}$) as a function of whether the last three trials were successes or failures. That is, we fit $\Delta X_t = w_0 + w_1 f_{t-1} + w_2 f_{t-2} + w_3 f_{t-3}$, where f_t is 1 if trial t was a failure and 0 for success. Note that w_0 reflects the change in reach when there were no failures in the previous three trials. This decreased with age and may represent decreasing sensorimotor noise (w_0: 100 out of 111 participants significant at p=0.05; correlation with age R^2 = 0.122, F = 15.2, p < 0.001). w_1, w_2 and w_3 reflect the contribution of failing on the previous trial, two and three trials ago, respectively to the change in reach. The change in reach after one failure increased with age (w_1: n = 66 out of 111 participants significant; correlation with age R^2 = 0.139, F = 17.6, p < 0.001). The effect of failure for two and three trials back were mostly not significant (w_2: 20 out of 111 participants significant; correlation with age R^2 = 0.0166, F = 1.83, p = 0.178; w_3: 17 out of 111 participants significant; correlation with age R^2 = 0.119, F = 14.7, p < 0.001). For the adults, the average of all of the data points is plotted as a horizontal line.

bioRxiv preprint doi: https://doi.org/10.1101/2024.07.09.602665; this version posted July 9, 2024. The copyright holder for this preprint (which was not certified by peer review) is the author/funder, who has granted bioRxiv a license to display the preprint in perpetuity. It is made available under aCC-BY 4.0 International license.

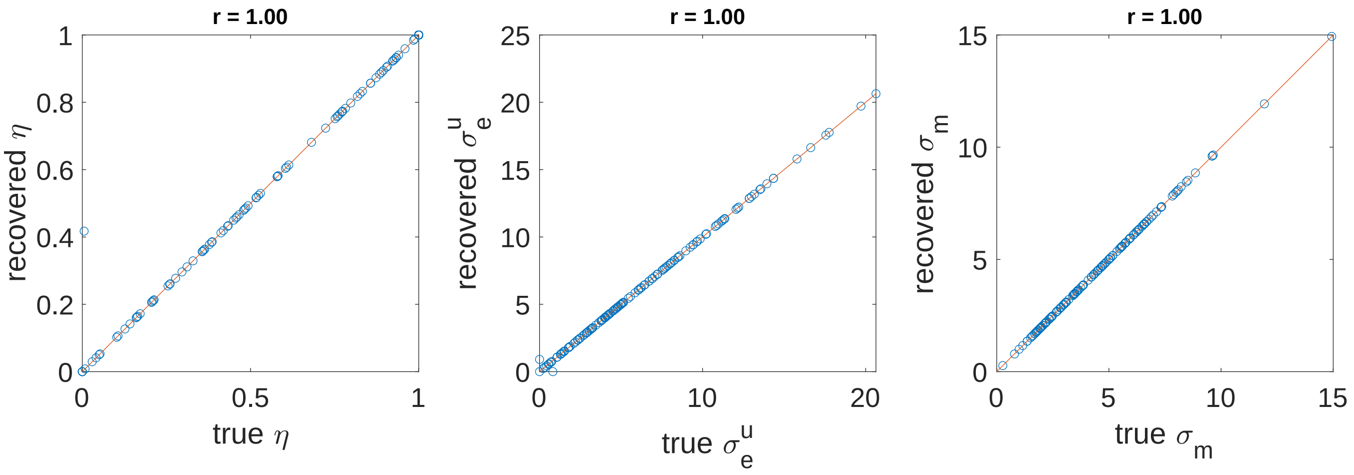

Supp. Fig. 7. Model parameter recovery. The recovered vs. true parameters for synthetic data generated by the model (for 100 learning trials) and then fit. Correlations are shown above the plots.

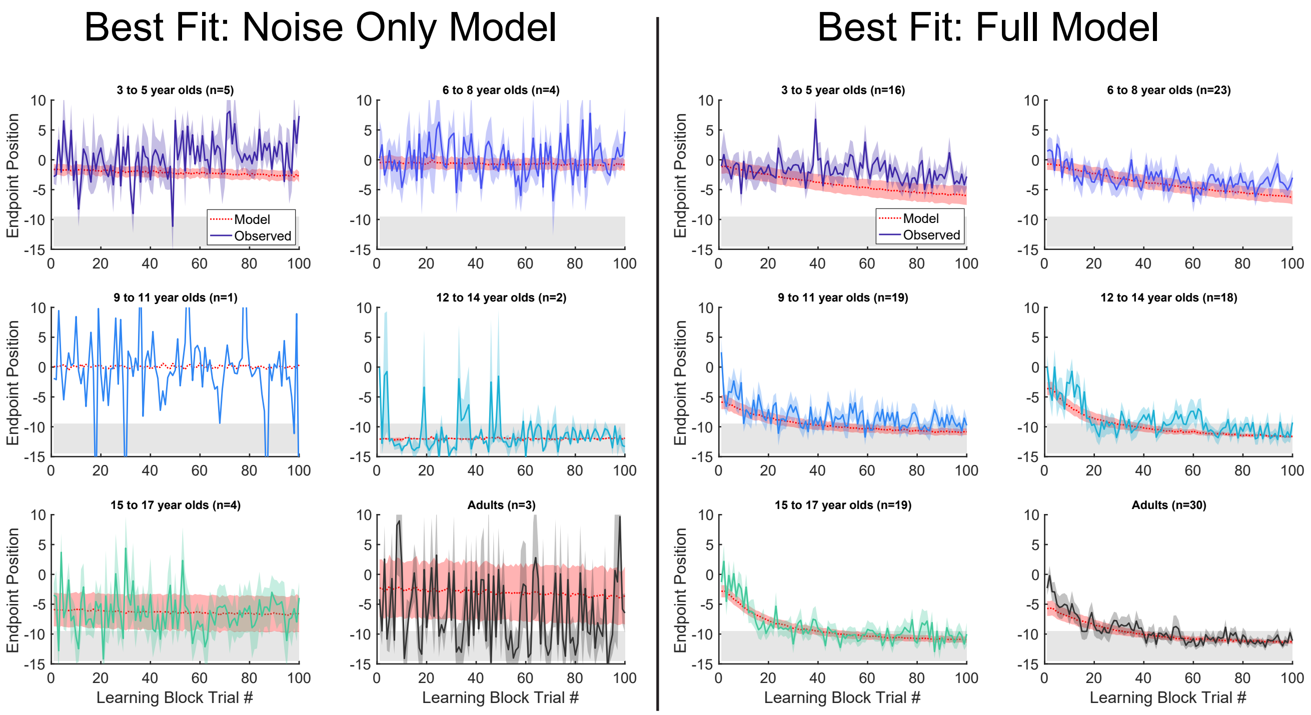

Supp. Fig. 8. Comparison of significant models. Some participants in each age bin were best fit with the noise only model (left) compared to the full model (right). When removing the participants who were best fit with the noise only model, the same age related trends in learning remained as depicted in Fig. 3. Children age 3 to 8 years old show poor learning compared to older participants.

bioRxiv preprint doi: https://doi.org/10.1101/2024.07.09.602665; this version posted July 9, 2024. The copyright holder for this preprint (which was not certified by peer review) is the author/funder, who has granted bioRxiv a license to display the preprint in perpetuity. It is made available under aCC-BY 4.0 International license.

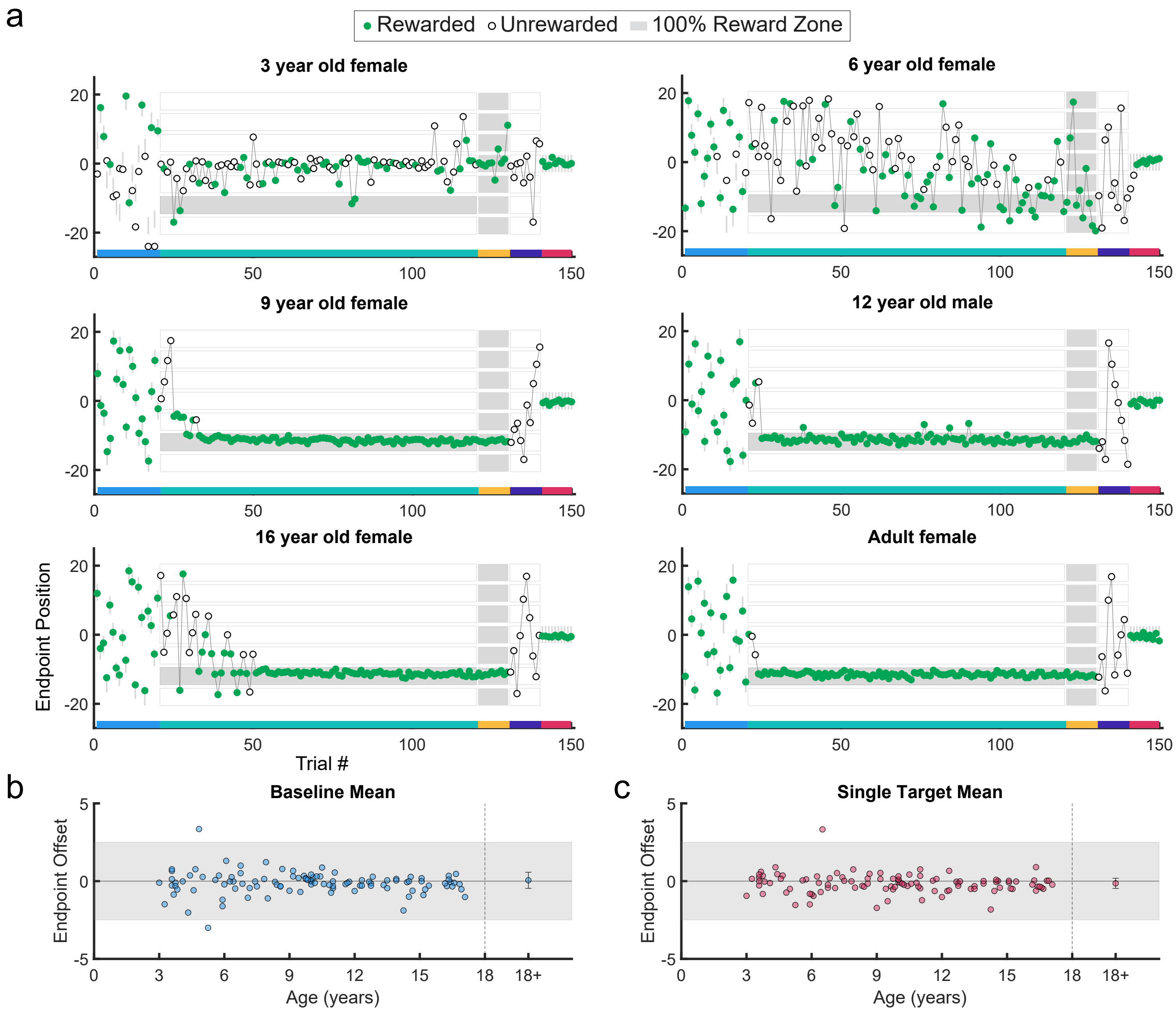

Supp. Fig. 9. Example behavior, and discrete target performance in the discrete probabilistic task. Same format as Fig. 2.

bioRxiv preprint doi: https://doi.org/10.1101/2024.07.09.602665; this version posted July 9, 2024. The copyright holder for this preprint (which was not certified by peer review) is the author/funder, who has granted bioRxiv a license to display the preprint in perpetuity. It is made available under aCC-BY 4.0 International license.

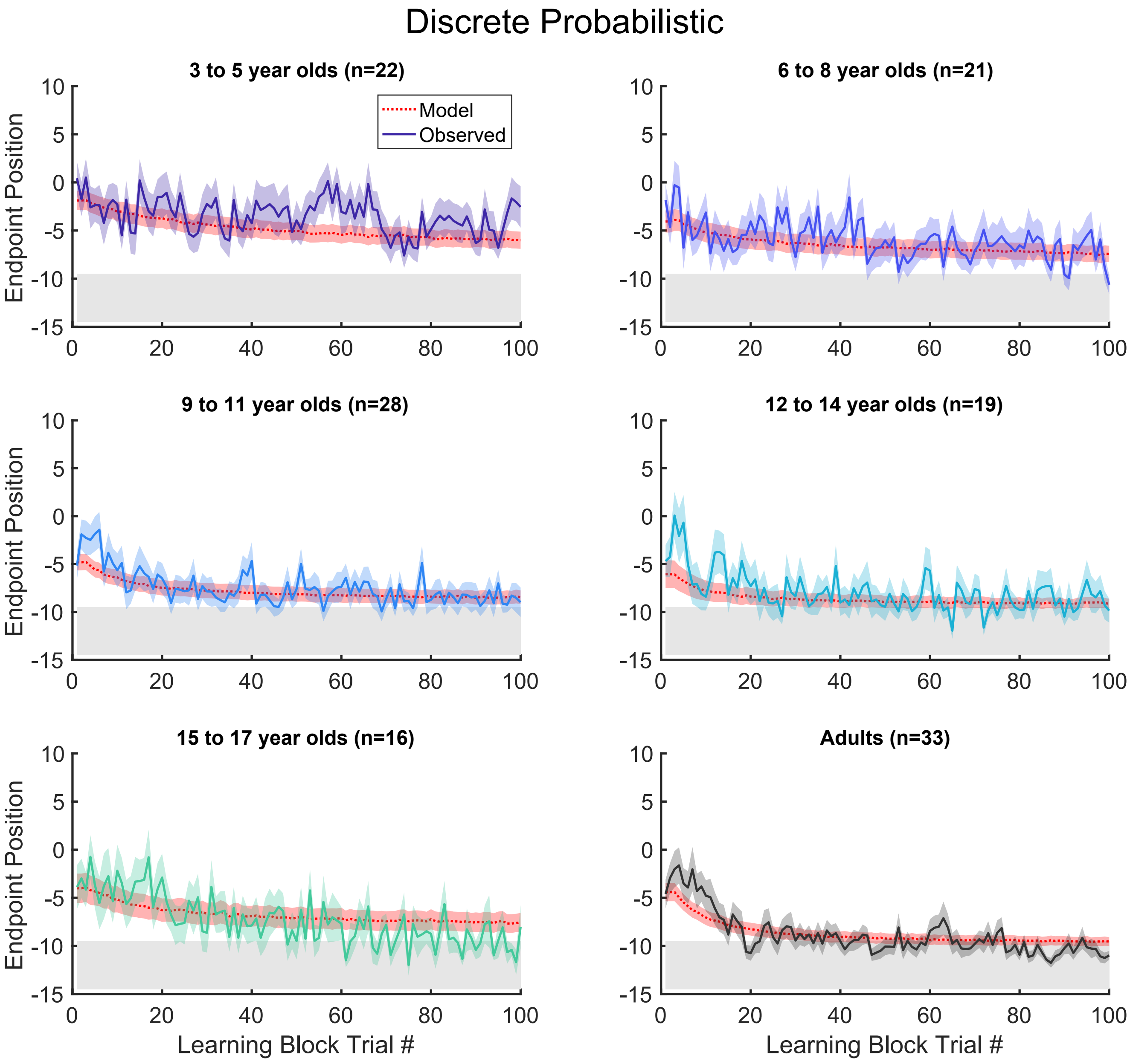

Supp. Fig. 10. Discrete probabilistic task learning block time series. Same format as Fig. 3.

bioRxiv preprint doi: https://doi.org/10.1101/2024.07.09.602665; this version posted July 9, 2024. The copyright holder for this preprint (which was not certified by peer review) is the author/funder, who has granted bioRxiv a license to display the preprint in perpetuity. It is made available under aCC-BY 4.0 International license.

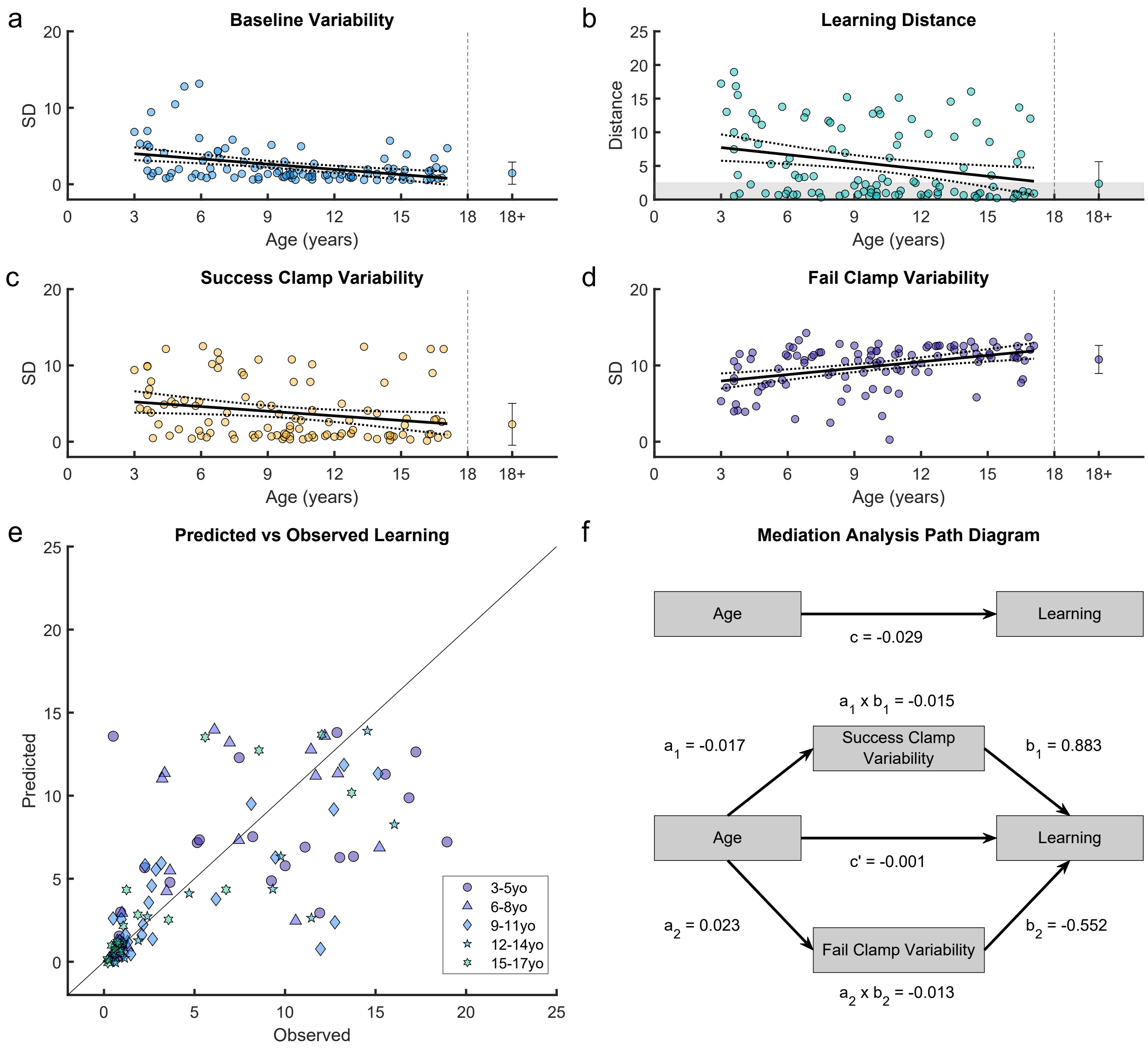

Supp. Fig. 11. Variability and learning in the discrete probabilistic task. Same format as Fig. 4.

bioRxiv preprint doi: https://doi.org/10.1101/2024.07.09.602665; this version posted July 9, 2024. The copyright holder for this preprint (which was not certified by peer review) is the author/funder, who has granted bioRxiv a license to display the preprint in perpetuity. It is made available under aCC-BY 4.0 International license.

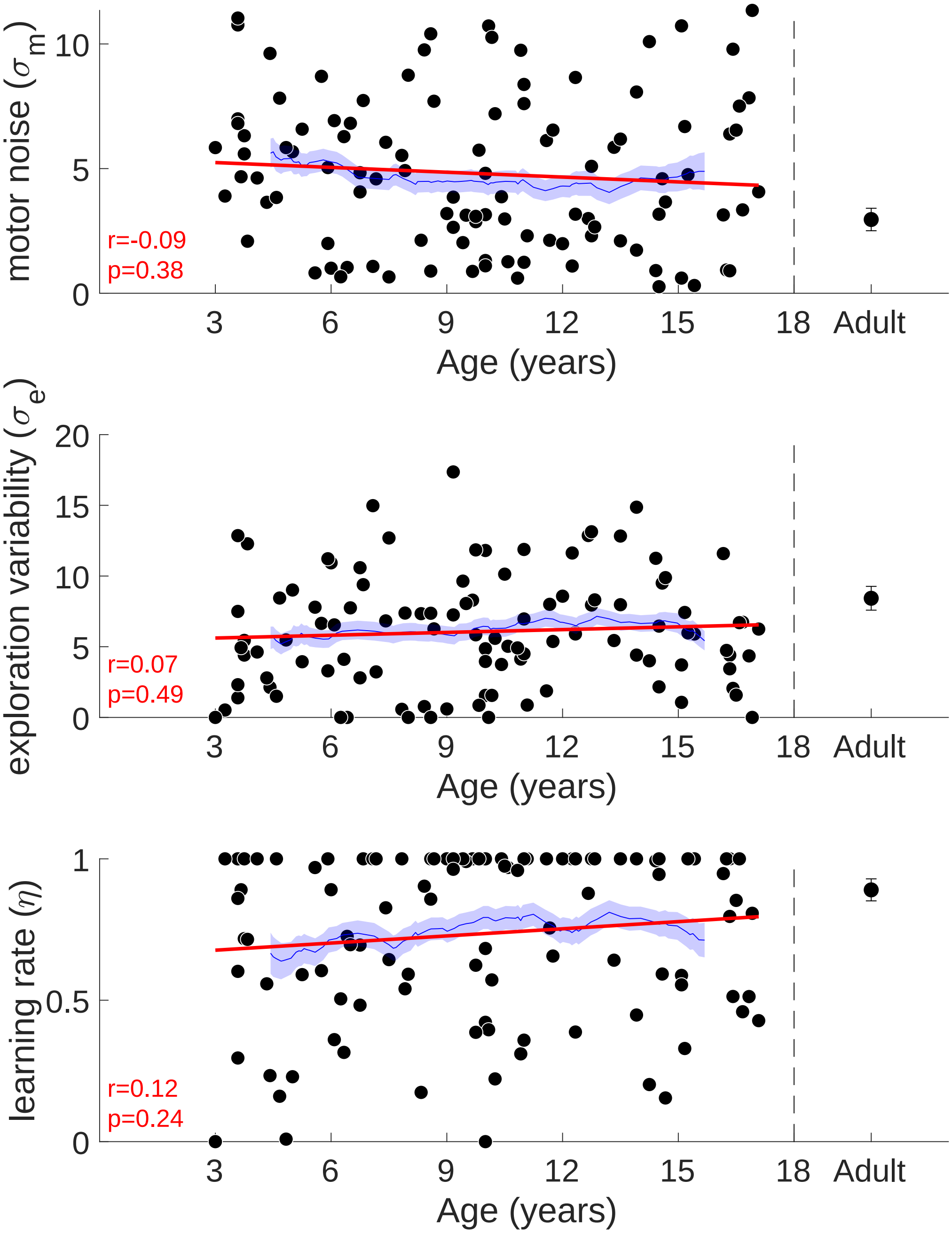

Supp. Fig. 12. Reinforcement learning model for the discrete probabilistic task. Panels in same format as Fig. 5b.

bioRxiv preprint doi: https://doi.org/10.1101/2024.07.09.602665; this version posted July 9, 2024. The copyright holder for this preprint (which was not certified by peer review) is the author/funder, who has granted bioRxiv a license to display the preprint in perpetuity. It is made available under aCC-BY 4.0 International license.

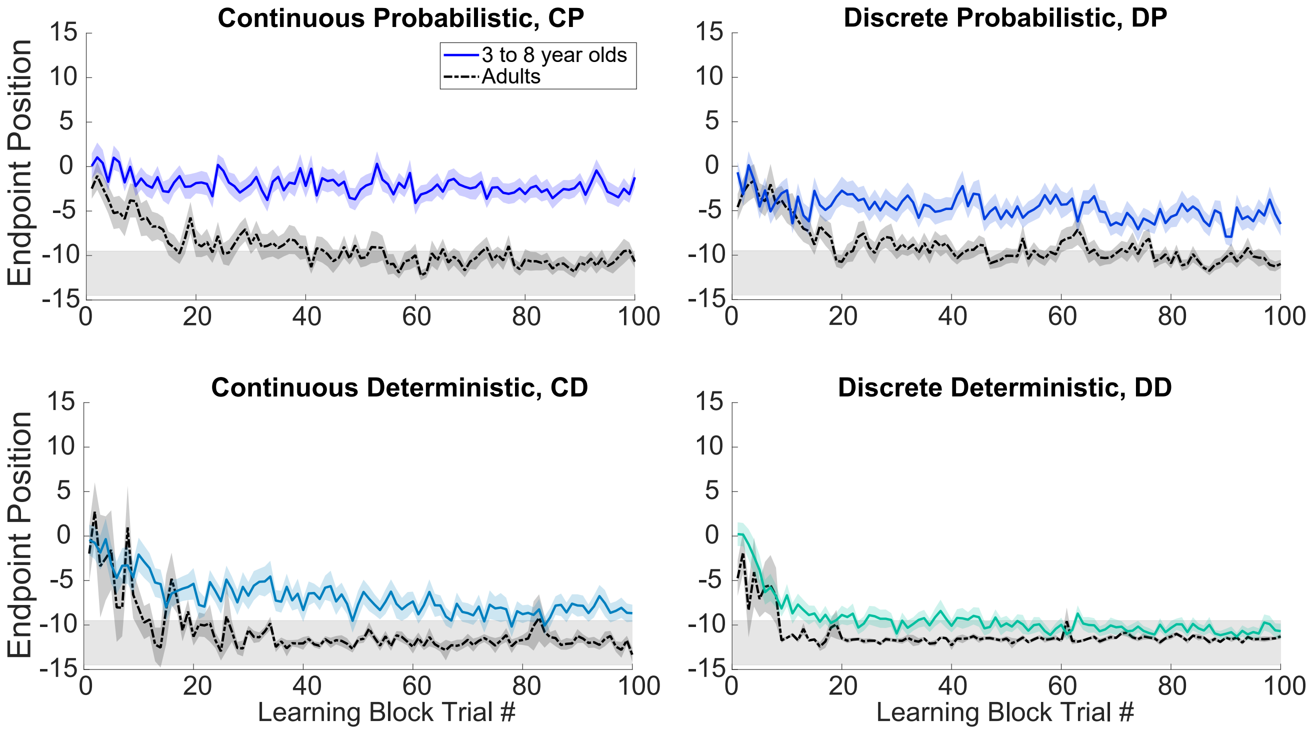

Supp. Fig. 13. Adult and 3 to 8-year-old children performance on all four tasks. Panels in same format as Fig. 6a.
